# Characterizing the interplay between gene nucleotide composition bias and splicing

**DOI:** 10.1186/s13059-019-1869-y

**Published:** 2019-11-29

**Authors:** Sébastien Lemaire, Nicolas Fontrodona, Fabien Aubé, Jean-Baptiste Claude, Hélène Polvèche, Laurent Modolo, Cyril F. Bourgeois, Franck Mortreux, Didier Auboeuf

**Affiliations:** 1 Laboratory of Biology and Modelling of the Cell, Univ Lyon, ENS de Lyon, Univ Claude Bernard, CNRS UMR 5239, INSERM U1210, 46 Allée d’Italie Site Jacques Monod, F-69007 Lyon, France; 2 CECS, I-Stem, F-91100 Corbeil-Essonnes, France; 3 LBMC Biocomputing Center, CNRS UMR 5239, INSERM U1210, 46 Allée d’Italie Site Jacques Monod, F-69007 Lyon, France

**Keywords:** Splicing, Genomic, Chromatin organization, Nucleotide composition bias

## Abstract

**Background:**

Nucleotide composition bias plays an important role in the 1D and 3D organization of the human genome. Here, we investigate the potential interplay between nucleotide composition bias and the regulation of exon recognition during splicing.

**Results:**

By analyzing dozens of RNA-seq datasets, we identify two groups of splicing factors that activate either about 3200 GC-rich exons or about 4000 AT-rich exons. We show that splicing factor–dependent GC-rich exons have predicted RNA secondary structures at 5′ ss and are dependent on U1 snRNP–associated proteins. In contrast, splicing factor–dependent AT-rich exons have a large number of decoy branch points, SF1- or U2AF2-binding sites and are dependent on U2 snRNP–associated proteins. Nucleotide composition bias also influences local chromatin organization, with consequences for exon recognition during splicing. Interestingly, the GC content of exons correlates with that of their hosting genes, isochores, and topologically associated domains.

**Conclusions:**

We propose that regional nucleotide composition bias over several dozens of kilobase pairs leaves a local footprint at the exon level and induces constraints during splicing that can be alleviated by local chromatin organization at the DNA level and recruitment of specific splicing factors at the RNA level. Therefore, nucleotide composition bias establishes a direct link between genome organization and local regulatory processes, like alternative splicing.

## Introduction

Most eukaryotic genes comprise both exons and introns. Introns are defined at their 5′-end by the 5′ splicing site (ss), which interacts with the U1 snRNA, and at their 3′-end, by the branch point (BP; recognized by SF1), the polypyrimidine (Py) tract (recognized by U2AF2 or U2AF65), and the 3′ ss (recognized by U2AF1 or U2AF35) [1]. SF1 and U2AF2 allow the recruitment of the U2 snRNP, which contains the U2 snRNA that interacts with the BP [1]. Recent large-scale experiments have demonstrated that RNA secondary structures frequently occur in the vicinity of splicing sites and gene-by-gene analyses have demonstrated that RNA structures play a direct role in splicing regulation [2]. For example, secondary structures at the 5′ ss can hinder the interactions between the 5′ ss and U1 snRNA and this structure-dependent constraint can be relaxed by RNA helicases, such as DDX5 and DDX17 [3–5]. Meanwhile, secondary structures at the 3′-end of short introns can replace the need for U2AF2 [6]. Splicing signals are short degenerate sequences, and exons are much smaller than introns. How then are exons precisely defined? How are bona fide splicing signals distinguished from pseudo-signals or decoy signals? These questions have been intensively researched but still remain open.

Many (if not all) exons require a variety of splicing factors to be defined. Splicing factors that belong to different families of RNA binding proteins, such as the SR and hnRNP families, bind to short degenerate motifs either in exons or introns of pre-mRNAs [7]. Splicing factor binding sites are low-complexity sequences comprising either the same nucleotide or dinucleotide [8–10]. Splicing factors modulate the recruitment of different spliceosome-associated components [7, 11].

Spliceosome assembly and the splicing process occur mostly during transcription [11, 12]. In this setting, the velocity of RNA polymerase II (RNAPII) influences exon recognition in a complex manner, as speeding up transcription elongation can either enhance or repress exon inclusion [13]. RNAPII velocity is in turn influenced by the local chromatin organization, such as the presence of nucleosomes [11, 12]. Nucleosomes are preferentially positioned on exons because exons have a higher GC content than introns, which increases DNA bendability [14–17]. In addition, Py tracts (mostly made of Ts) upstream of exons may form a nucleosome energetic barrier [14–17]. Nucleosomes influence splicing by slowing down RNAPII in the vicinity of exons and by modulating the local recruitment of splicing regulators [11, 12]. Indeed, depending on their specific chemical modifications (e.g., methylation), histone tails can interact directly or indirectly with splicing factors [18]. Therefore, exon recognition during the splicing process depends on a complex interplay between signals at the DNA level (e.g., nucleosome positioning) and signals at the RNA level (e.g., splicing factor binding sites).

Genes are not randomly organized across a genome, and nucleotide composition bias over genomic regions of varying lengths plays an important role in genome organization at multiple genomic scales. For example, isochores are large genomic regions (≥ 30 kbps) with a uniform GC content that differs from adjacent regions [19–21]. Isochores can be classified into five families, ranging from less than 37% of GC content to more than 53% [19–21]. GC-rich isochores have a higher density of genes than AT-rich isochores, and genes in GC-rich isochores contain smaller introns than genes in AT-rich isochores [22–24]. In addition, to be associated with specific gene features (e.g., intron size), the density of GC nucleotides in genes has consequences on splicing site sequences and on the splicing process [25]. For example, it has been proposed that splicing of short introns in a GC-rich context may occur through the intron definition model, while the splicing of large introns in an AT-rich context may occur through the exon definition model [11, 26]. Collectively, these observations support a model in which the gene architecture and gene nucleotide composition bias (e.g., GC or AT content) influence local processes at the exon level, such as nucleosome positioning and intron removal. As exon recognition also depends on the binding to the pre-mRNAs of splicing factors that interact with compositionally biased sequences, one interesting possibility is that the nature of these splicing factors depends at least in part on the gene nucleotide composition bias. In this setting, we have recently reported that exons regulated by different splicing factors have different nucleotide composition bias [27].

Here, we have investigated the relationship between the splicing process, gene nucleotide composition bias, and chromatin organization at both the local and global levels. We initially identified sets of exons activated by different splicing factors and then demonstrated that analyzing the nucleotide composition bias provided a better understanding of the interplay between chromatin organization and splicing-related features, which collectively affect exon recognition. We propose that nucleotide composition bias not only contributes to the 1D and 3D genome organization, but has also local consequences at the exon level during the splicing process.

## Results

### Splicing factor–dependent GC-rich and AT-rich exons

Publicly available RNA-seq datasets generated after knocking down or overexpressing individual splicing factors across different cell lines were analyzed [28–43] (Additional file 1: Table S1). Using our recently published FARLINE pipeline, which allows the inclusion rate of exons from RNA-seq datasets to be quantified [44], we defined the sets of exons whose inclusion is activated by each of the 33 splicing factors that were analyzed (Additional file 2: Table S2). We focused on splicing factor–activated exons to uncover the splicing-related features characterizing exons whose recognition depends on at least one splicing factor. We identified 10,707 exons that were activated by at least one splicing factor from the 93,680 exons whose inclusion rate was quantified by FARLINE across all datasets (see the “Materials and methods” section).

**Fig. 1:**
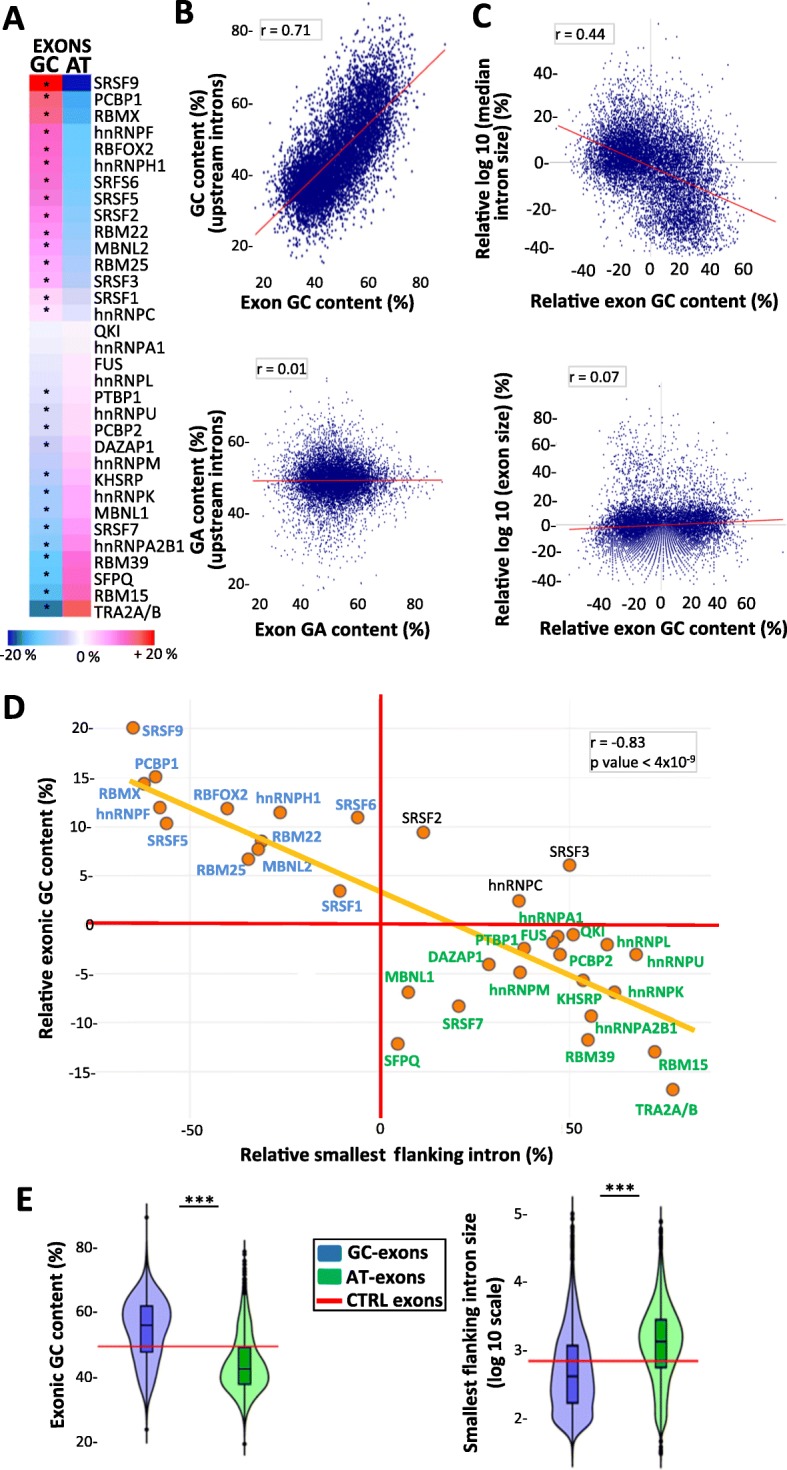
Splicing factor–dependent GC-rich and AT-rich exons. **a** Heatmaps representing the relative median frequency of GC and AT nucleotides in sets of splicing factor–activated exons, as compared to the median values computed from control exons. “*” corresponds to Student’s test FDR < 0.05. **b** Correlation between the GC or GA content (upper or lower panel, respectively) of splicing factor–activated exons and the GC or GA content, respectively, of their upstream intron; *r* = Pearson correlation coefficient. **c** Correlation between the relative GC content of splicing factor–activated exons and the size of their flanking introns (upper panel) or the exon size (lower panel); *r* = Pearson correlation coefficient. **d** The *x*-axis represents the relative median size of the smallest intron flanking splicing factor–activated exons, as compared to the median size of human introns. The *y*-axis represents the relative median GC content of splicing factor–activated exons, as compared to the median GC frequency of control exons; *r* = Pearson correlation coefficient. **e** Violin plots representing the GC content (%) of GC exons and AT exons (left panel), and the logarithmic nucleotide size of the smallest intron flanking GC exons and AT exons (right panel). The red lines indicate the median values computed for control exons. “***” corresponds to Wilcoxon’s test *P* < 10^− 16^ when comparing GC exons to AT exons

As expected, splicing factor–activated exons had weaker 3′- and/or 5′-splicing site (ss) scores as compared to the median score of human exons (Additional file 3: Figure S1). We computed the nucleotide composition of each splicing factor–activated exon (Additional file 3: Figure S2). Note that we systematically refer to both thymine and uracil as “T” to simplify our goal of analyzing sequence-dependent features at both the DNA and RNA levels. In addition, values obtained from splicing factor–activated exons were normalized by the median values measured for human coding exons used as a set of control exons, in order to represent results in a consistent way. Sets of exons activated by different splicing factors had a different proportion of GCs as compared to the median values of control exons (Fig. 1a, Additional file 3: Figure S3a). Interestingly, the GC content of splicing factor–activated exons positively correlated with the GC content of their flanking introns (Fig. 1b, upper panel, *P* value < 10^−16^ and Additional file 3: Figure S3d). Accordingly, splicing factor–activated GC- and AT-rich exons were flanked by GC- and AT-rich intronic sequences, respectively (Additional file 3: Figure S3b, c). This result is in agreement with previous observations [45]. Of note, there was no correlation between the purine or pyrimidine content of exons and that of their flanking introns (Fig. 1b, lower panel and see the “Discussion” section).

Size analysis of introns flanking splicing factor–activated exons revealed that different sets of splicing factor–activated exons were flanked by introns that were either smaller or larger than the median size of human introns (Additional file 3: Figure S4a, b). Interestingly, there was a negative correlation between the GC content of exons and the size of their flanking introns (Fig. 1c, upper panel, *P* value < 10^−16^), as previously reported [24], but not between exon GC content and exon size (Fig. 1c, lower panel). Based on these observations, we defined two groups of exons. The GC-exon group depends on splicing factors activating GC-rich exons that are flanked by small introns (Fig. 1d, in blue), while the AT-exon group depends on splicing factors activating AT-rich exons that are flanked by large introns (Fig. 1d, in green). We excluded for further analyses exons regulated by SRSF2, SRSF3, or hnRNPC, as these splicing factors regulate GC-rich exons flanked by relatively large introns, as well as exons belonging to both groups (see the “Materials and methods” and “Discussion” sections). We next analyzed different splicing-related features by comparing 3182 GC exons to 4045 AT exons, representing two populations of exons that (i) differ in terms of both GC content and flanking intron size and (ii) are activated by distinct splicing factors (Fig. 1d, e and Additional file 2: Table S2).

### Nucleotide composition bias and splicing-related features

**Fig. 2:**
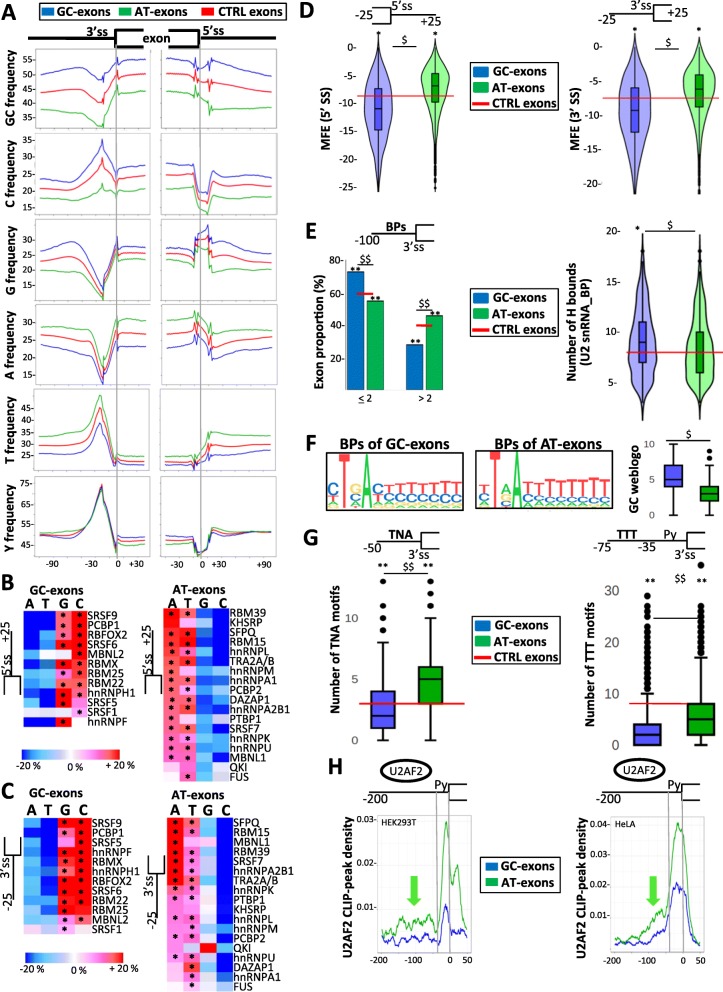
Nucleotide composition bias and splicing-related features. **a** Nucleotide frequency (%) maps in different sets of exons and their flanking intronic sequences. **b** Heatmap representing the average frequency (%, as compared to control exons) of A, T, G, or C nucleotides in a window of 25 nucleotides downstream of GC exons (left panel) or AT exons (right panel). “*” corresponds to Wald’s test FDR < 0.05. **c** Heatmap representing the average frequency (%, as compared to control exons) of A, T, G, or C nucleotides in a window of 25 nucleotides upstream of GC exons (left panel) or AT exons (right panel). “*” corresponds to Wald’s test FDR < 0.05. **d** Minimum free energy (MFE) at the 5′ ss (left panel) and the 3′ ss (right panel) of GC exons or AT exons. MFE was computed using 25 nucleotides within exons and 25 nucleotides within introns. The red lines indicate the median values calculated for control exons. “^$^” and “*” correspond to Tukey’s test FDR < 10^−16^ when comparing GC exons to AT exons or when comparing GC exons or AT exons to control exons, respectively. **e** Proportion (%) of GC exons or AT exons with at least two or more predicted BPs in a window of 100 nucleotides in their upstream intron (left panel). Number of hydrogen bonds measured between the U2 snRNA and the BP sequence found in the 25 nucleotides upstream of GC exons and AT exons (right panel). The red lines indicate the median values calculated for control exons. “**” and “^$$^” correspond to *χ*^2^ test *P* < 10^−13^ when comparing GC exons to AT exons or when comparing GC exons or AT exons to control exons, respectively. “^$^” and “*” correspond to Tukey’s test *P* < 0.02 when comparing GC exons to AT exons and when comparing GC exons to control exons, respectively. **f** Weblogos generated using sequences flanking the BPs with the best score in a 25 nucleotide-long window upstream of GC exons or AT exons and the boxplot resuming their GC content. “^$^” corresponds to Tukey’s test FDR < 10^−16^. **g** Boxplot representing the number of TNA sequences within the last 50 nucleotides of the upstream introns of GC exons and AT exons (left panel). Boxplot representing the number of T-rich low-complexity sequences in a window between positions − 35 and − 75 upstream the 3′ ss of GC exons and AT exons (right panel). The red lines indicate the median values calculated for control exons. “^$$^” and “**” correspond to Tukey’s FDR < 10^−16^ when comparing GC exons to AT exons and when comparing GC exons or AT exons to control exons, respectively. **h** Density of peaks obtained from publicly available U2AF2-CLIP datasets generated from HEK293T (left panel) or HeLa (right panel) cells and mapped upstream of GC exons and AT exons. The green arrows indicate peaks that mapped upstream of the Py tract

We found that exons and their flanking intronic sequences had similar nucleotide composition biases when considering both whole intronic sequences (Fig. 1b, upper panel, and Additional file 3: Figure S3d) or intronic sequences located just upstream or downstream exons (Fig. 2a–c). For example, 25 or 100 nucleotide-long intronic sequences that flank GC exons had a higher frequency of G and/or C nucleotides as compared to intronic sequences flanking AT exons (Fig. 2a–c). A higher GC content was associated with a lower minimum free energy measured by using the Vienna RNA package [46] in a 50 nucleotide-long window centered at the 5′ ss, when comparing GC exons to both control exons and AT exons (Fig. 2d, left panel). This suggests a higher stability of base pairing between complementary sequences and that the 5′ ss of GC exons are more likely to be embedded in stable secondary structures than AT exons. A similar feature was observed at the 3′ ss of GC exons when compared to AT exons (Fig. 2d, right panel).

GC exons were impoverished in Ts but enriched in Cs just upstream of their 3′ ss as compared to control exons (Fig. 2a, c). Therefore, the pattern of pyrimidines upstream of GC exons was similar to that of control exons (Fig. 2a), although the high density of Cs upstream of GC exons may have consequences on Py-tract recognition by U2AF2 (see below). Meanwhile, AT exons had a higher frequency of As upstream of their 3′ ss as compared to GC exons or control exons (Fig. 2a, c). We tested whether this was associated with a larger number of potential BP sites, which often contain As by using the pipeline developed by Corvelo et al. [47–49]. Indeed, a higher proportion of AT exons had more than two predicted BPs in their upstream intronic sequence, as compared to GC exons or control exons (Fig. 2e, left panel). Further, predicted BPs upstream of GC exons were embedded in sequences that contained a slightly higher proportion of Cs as compared to AT exons (Fig. 2f). The interaction between the BP and U2 snRNA was reported to be more stable when the BP is embedded in GC-rich sequences [47–49]. Accordingly, the number of hydrogen bonds between BP sites and U2 snRNA was higher for GC exons than for AT exons (Fig. 2e, right panel).

As there was a higher frequency of As and Ts upstream of AT exons as compared to control exons (Fig. 2a, c), we investigated whether this may interfere with the number of potential binding motifs for SF1 (which binds to UNA motifs) and U2AF2 (which binds to U-rich motifs) [1]. As shown in Fig. 2g (left panel), AT exons contained a larger number of TNA motifs upstream of their 3′ ss as compared to GC exons. In addition, AT exons contained a larger number of low-complexity sequences made of three Ts within a four-nucleotide window upstream of the Py-tract as compared to GC exons (Fig. 2g, right panel). Supporting the biological relevance of this observation, the analysis of U2AF2 CLIP-seq datasets revealed a higher U2AF2-related signal upstream of AT exons, as compared to GC exons (Fig. 2h), which extended upstream of the Py tract of AT exons (green arrows). This is consistent with the differential pattern of T and C frequency between the two sets of exons (Fig. 2a) and with the fact that Us provide higher binding affinity to U2AF2 than Cs [50].

### Nucleotide composition bias and dependency for specific spliceosome components

**Fig. 3:**
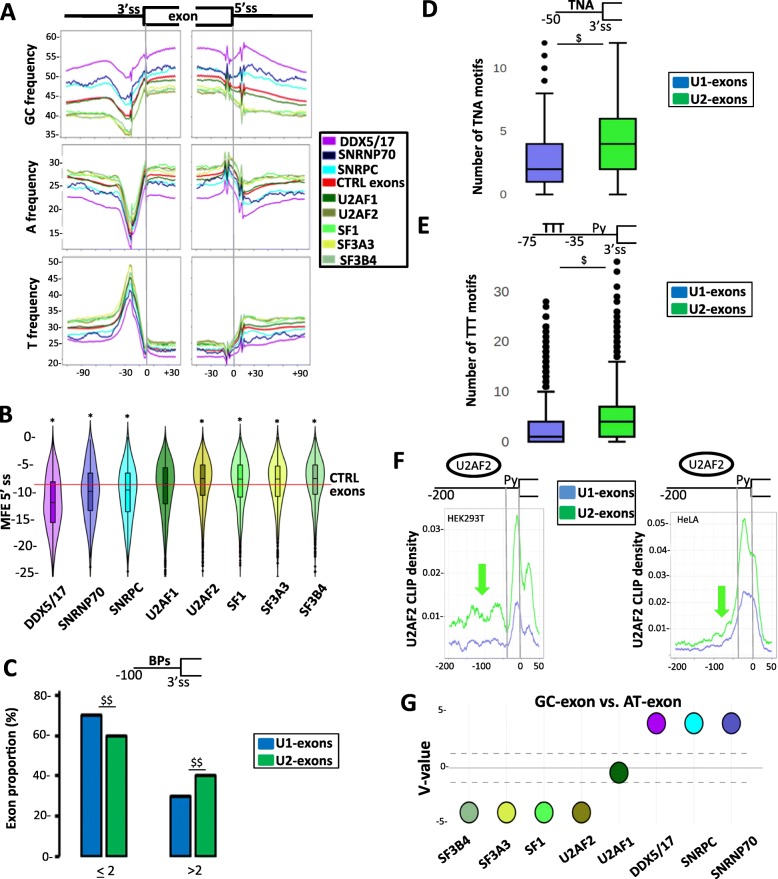
Nucleotide composition bias and dependency for specific spliceosome components. **a** Nucleotide frequency (%) maps in different sets of exons and their flanking intronic sequences. **b** Minimum free energy (MFE) at the 5′ ss of sets of exons activated by different spliceosome-associated factors. MFEs were computed using 25 nucleotides within the exons and 25 nucleotides within the intron. The red line indicates the median values calculated for control exons. “*” corresponds to Student’s test FDR < 0.02 when comparing a set of exons activated by a spliceosome-associated factor to control (CTRL) exons. **c** Proportion (%) of exons activated by U1 snRNP–associated factors (U1 exons) or by U2 snRNP–associated factors (U2 exons) with two or more predicted BPs in a window corresponding to the last 100 nucleotides in their upstream intron. “^$$^” corresponds to *χ*^2^ test *P* < 10^−16^ when comparing U1 exons to U2 exons. **d** Boxplot of the number of TNA sequences in the last 50 nucleotides upstream introns of U1 exons and U2 exons. “^$^” corresponds to Wald’s test *P* value < 10^−16^ when comparing U1 exons to U2 exons. **e** Boxplot of the number of T-rich low-complexity sequences in a window between positions − 35 and − 75 upstream the 3′ ss of U1 exons and U2 exons. “^$^” corresponds to Wald’s test *P* value < 10^−16^ when comparing U1 exons to U2 exons. **f** Density of peaks obtained from publicly available U2AF2-CLIP datasets generated from HEK293T (left panel) or HeLa (right panel) cells and mapped upstream of U1 exons or U2 exons. The green arrows indicate peaks mapping upstream of the Py tract. **g** The *V* value is a representation of a *P* value calculated by comparing the proportion of GC exons and AT exons activated by individual spliceosome-associated factors (see the “Materials and methods” section). A *V* value above the dotted line that corresponds to log10 (0.05) is statically significant

To investigate the interplay between nucleotide composition bias and the dependency of exons on specific spliceosome-associated factors, we analyzed publicly available RNA-seq datasets generated after knocking down a variety of spliceosome-associated factors [28, 51–54] (Additional file 1: Table S1 and Additional file 2: Table S2). Exons that were skipped after depletion of SNRPC or SNRNP70 (two components of the U1 snRNP) were in a more GC-rich environment as compared to control exons (Fig. 3a). Likewise, exons that were skipped upon the depletion of the DDX5 and DDX17 RNA helicases (DDX5/17), which enhance exon inclusion by favoring U1 snRNP binding to highly structured 5′ ss [4, 5, 55], were in a GC-rich environment (Fig. 3a). In addition, the 5′ ss of exons dependent on SNRPC, SNRNP70, or DDX5/17 were predicted to be embedded in stable secondary structures as compared to control exons (Fig. 3b).

Exons skipped after depletion of SF1, U2AF2, SF3A3, or SF3B4 (but not U2AF1) that recognize splicing signals at 3′-ends of introns were in an AT-rich environment as compared to control exons (Fig. 3a). In addition, a larger proportion of U2-exons—that is, those activated by the U2 snRNP–associated factors including SF1, U2AF2, SF3A3 or SF3B4—contained more than two predicted BPs in their upstream intron as compared to U1 exons (e.g., those activated by SNRPC, SNRNP70, and/or DDX5/17) (Fig. 3c). In addition, U2 exons contained a larger number of SF1- and U2AF2-binding sites in their upstream intron as compared to U1 exons (Fig. 3d, e). In agreement with the T-frequency pattern (Fig. 3a), a broader U2AF2-derived signal was observed when comparing U2 exons to U1 exons (Fig. 3f).

To summarize, GC exons were predicted to have stable secondary structures at their 5′ ss (Fig. 2a, d), and the nucleotide composition bias and splicing-related features of U1 exons were similar to those of GC exons (Fig. 3a, b). Additionally, the increased frequency of As and Ts upstream of AT exons (Fig. 2a) was associated with increased numbers of potential decoy signals, and the nucleotide composition bias and splicing-related features of U2 exons were similar to those of AT exons (Fig. 3a, c–f). We therefore hypothesized that GC exons were more sensitive to U1 snRNP- than to U2 snRNP–associated factors, in contrast to AT exons. Accordingly, 28% of GC exons and 14% of AT exons were regulated by U1-related factors, while 27% of AT exons and 17% of GC exons were regulated by U2-related factors. In addition, GC exons were more likely to be affected by SNRPC, SNRNP70, or DDX5/17 depletion than AT exons, which were more likely to be affected by SF1, U2AF2, SF3A3, or SF3B4 depletion (Fig. 3g).

**Fig. 4:**
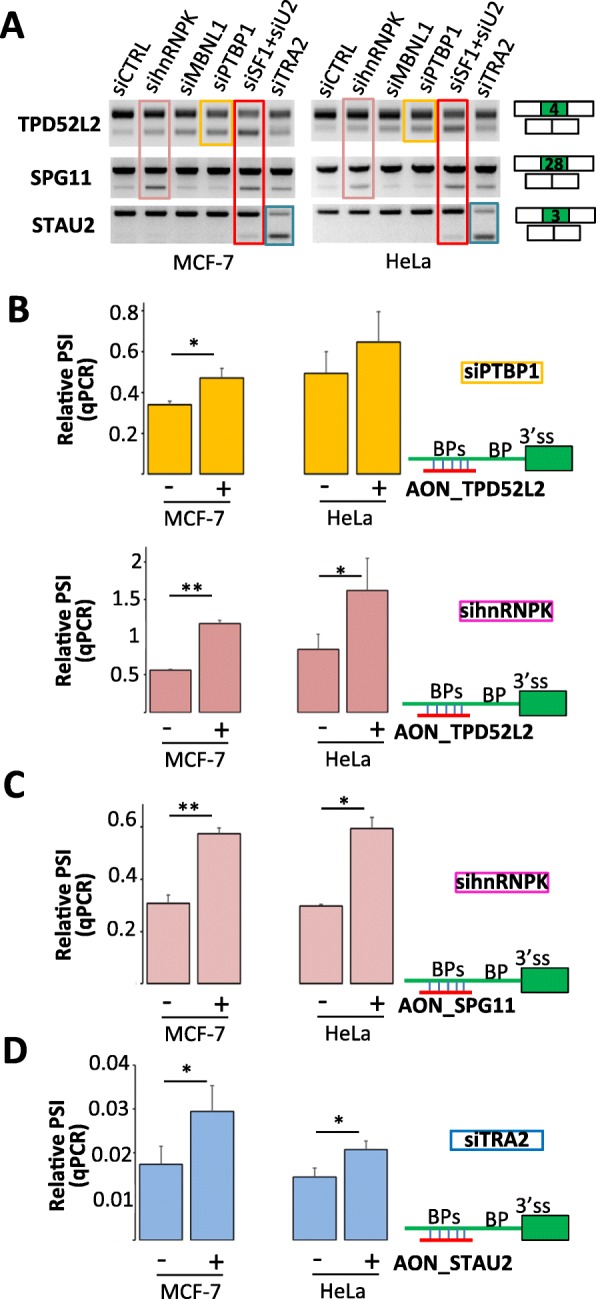
Splicing regulation by splicing factor–mimicking oligonucleotides. **a** MCF-7 and HeLa cell lines were transfected with siRNAs (as indicated), and the splicing pattern of the indicated exons was analyzed by RT-PCR. siU2 corresponded to a mix of siSF1 and siU2AF2. Each siRNA had a specific effect on the selected exons. **b** MCF-7 and HeLa cell lines were transfected with a control siRNA, siPTBP1 (upper panel), or sihnRNPK (lower panel) and a control AON (−) or AON_TDP52L2 (+) targeting supernumerary predicted BPs upstream of TPD52L2 exon 4. Relative PSI as measured by qPCR represents the inclusion/exclusion ratio normalized by the inclusion/exclusion ratio obtained in control cells (i.e., cells transfected with siCTRL and a control AON). Box plots represent the mean value (+ S.E.M.) of three independent experiments. “*” corresponds to *P* value< 0.05 and “**”corresponds to *P* value< 0.005 (Student’s *t* test). **c** As in **b** using sihnRNPK and AON_SPG11 targeting intronic sequences upstream SPG11 exon 28. *P value < 0.05 and **P value < 0.005 (Student’s *t* test). **d** As in **b** using siTRA2 and AON_STAU2 targeting intronic sequences upstream STAU2 exon 3

Several publications have already shown that the negative effect of GC-rich structures on 5′ splice site recognition can be reversed by the helicase activity of DDX5 and DDX17 [3–5, 55]. To challenge our prediction about the regulation of AT exons, we selected from the analyzed RNA-seq datasets three exons that were activated by SF1 and U2AF2, and we tested the effect of several AT-exon regulating factors such as PTBP1, MBNL1, hnRNPK, and TRA2 (Fig. 1d). As shown in Fig. 4a, the selected exons were more excluded in the absence of both SF1 and U2AF2 (siU2, Fig. 4a) and the depletion of PTBP1, MBNL1, hnRNPK, or TRA2 had a selective effect on the different exons (Fig. 4a and b–d). Interestingly, the co-transfection of 2′-O-methylated antisense RNA oligonucleotides (AONs) targeting potential decoy BPs restored exon inclusion (Fig. 4b–d). For example, the depletion of PTBP1 or hnRNPK induced the exclusion of TD52L2 exon 4, which was reversed by the AON_TDP52L2 (Fig. 4b). These results showed that AONs can compensate the absence of AT-exon regulating splicing factors and are in agreement with a role of splicing factors in “filling up” or compensate a “surplus” of splicing signals [48, 53, 56–60] (see the “Discussion” section).

### Nucleotide composition bias, gene features, and chromatin organization

**Fig. 5:**
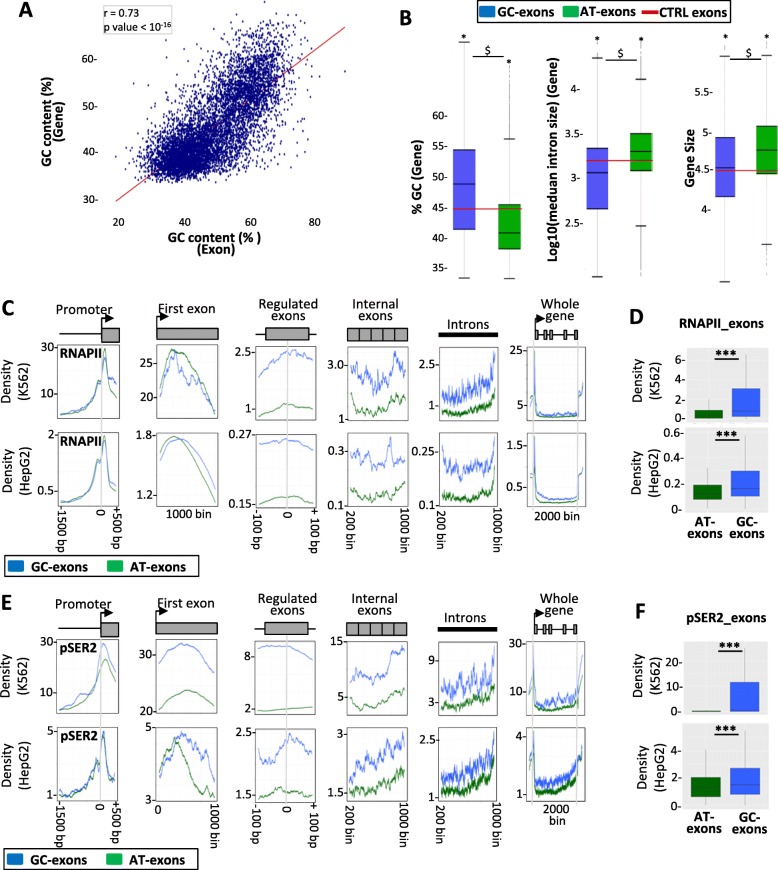
Nucleotide composition bias, gene features, and transcription. **a** Correlation between the GC content of splicing factor–activated exons and the GC content of their hosting genes; *r* = Pearson correlation coefficient. **b** Box plots representing the GC content (%, left panel), the intron size (middle panel), and the size (right panel) of genes hosting GC exons or AT exons. The red lines indicate the median values calculated for control exons. “^$^” and “*” correspond to Student’s test (left panel and right panel) and Wilcoxon’s test (middle panel) *P* value < 2 × 10^−4^ when comparing genes hosting GC exons and AT exons and when comparing genes hosting GC exons or AT exons to control genes, respectively. **c** Density of reads obtained after immunoprecipitation of RNAPII in K562 and HepG2 cell lines and then mapped to different parts of genes with GC exons or AT exons. **d** Box plots of the mean coverage by RNAPII of GC exons and AT exons. “***” corresponds to Wilcoxon’s test *P* < 10^−6^. **e** Density of reads obtained after immunoprecipitation of RNAPII phosphorylated at serine 2 (RNAPII-ser2) in K562 and HepG2 cell lines and then mapped to different parts of genes with GC exons or AT exons. **f** Box plots of the mean coverage of phosphorylated RNAPII at GC exons or AT exons. “***” corresponds to Wilcoxon’s test *P* < 10^−6^

As exons and their flanking intronic sequences have similar nucleotide composition biases (see Fig. 1b, Fig. 2a–c), we investigated whether the observed local nucleotide composition bias could be extended to the gene level. Indeed, there was a positive correlation between the GC content of exons and the GC content of their hosting gene (Fig. 5a), and GC exons and AT exons belong to GC- and AT-rich genes, respectively, as compared to all human genes (Fig. 5b, left panel). In addition, a negative correlation between the GC content of genes and the size of their introns was observed (Additional file 3: Figure S4c), as previously reported [24]. Accordingly, GC exons that are flanked by small introns (Fig. 1) belong to genes containing small introns (Fig. 5b, middle panel). Meanwhile, AT exons that are flanked by large introns (Fig. 1) belong to large genes containing large introns, as compared to all human genes (Fig. 5b, middle and right panels).

It has been reported that GC-rich genes are more expressed as compared to AT-rich genes [24, 61–63]. Remarkably, for genes comprising either AT exons or GC exons, the RNAPII density was similar at promoters and the first exon but was higher at exons and introns of genes hosting GC exons than genes hosting AT exons (Fig. 5c, d, Additional file 3: Figure S5a). A similar result was observed for the pattern of RNAPII phosphorylated at Ser2 (Fig. 5e, f), suggesting that the RNAPII content on genes hosting GC exons was likely to be productive.

**Fig. 6:**
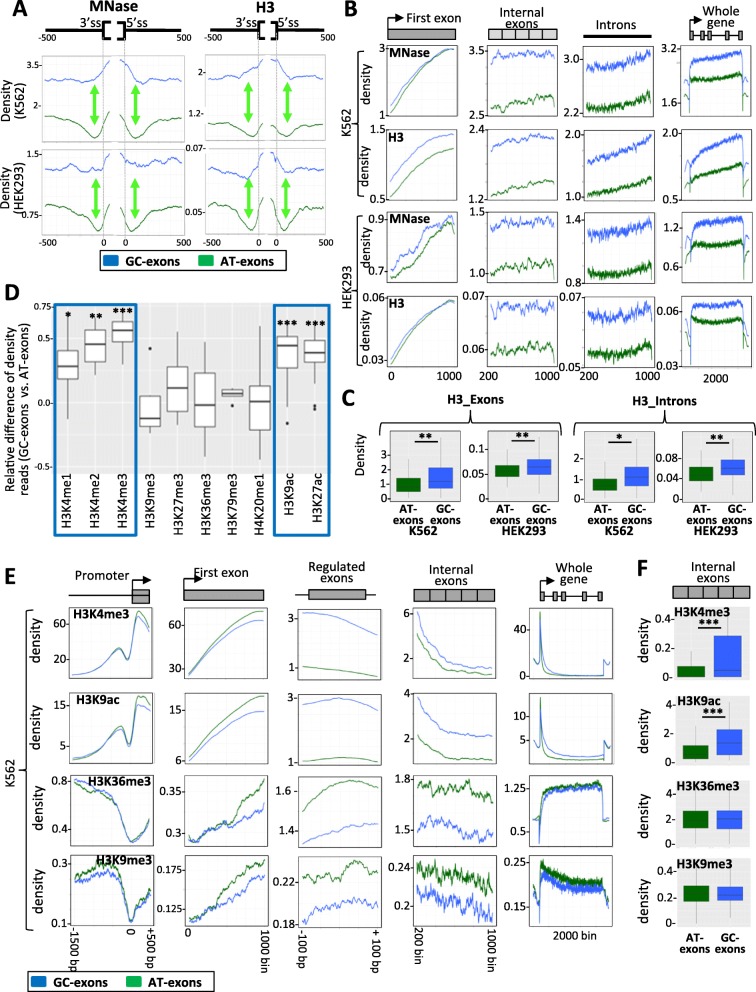
Nucleotide composition bias and chromatin organization. **a** Density of reads obtained after DNA treatment with MNase (left panels), or after immunoprecipitation of the H3 histone (right panels), in K562 and HEK293 cell lines and then mapped to GC exons or AT exons and their flanking introns. **b** Density of reads obtained after DNA treatment with MNase, or after immunoprecipitation of the histone H3, in K562 and HEK293 cell lines and then mapped to different parts of genes with GC exons or AT exons. **c** Box plots of the mean coverage of reads obtained after immunoprecipitation of the histone H3 from K562 or HEK293 cell lines and then mapped to exons and introns of genes hosting GC exons or AT exons. “*” corresponds to Wilcoxon’s test *P* < 0.05. “**” corresponds to Wilcoxon’s test *P* < 0.001. **d** Box plots representing the relative difference of density reads obtained after DNA immunoprecipitation using antibodies against different histone modifications (as indicated) and then mapped to GC exons or AT exons. Each box plot represents the values obtained from several publicly available datasets. “*” corresponds to Wilcoxon’s test *P* < 0.005; “**” corresponds to Wilcoxon’s test *P* < 0.001; “***” corresponds to Wilcoxon’s test *P* < 0.0001. **e** Density of reads obtained from the K562 cell line after immunoprecipitation of DNA using antibodies against different histone modifications (as indicated) and then mapped to different parts of genes with GC exons or AT exons. **f** Box plots of the mean coverage of reads obtained after DNA immunoprecipitation using antibodies against H3K4me3, H3K9ac, H3K36me3, or H3K9me3, and then mapped to exons of genes with GC exons or AT exons. “***” corresponds to Wilcoxon’s test *P* < 10^−6^

In addition, to be associated with gene expression level, the GC content is associated with nucleosome positioning (see the “Introduction” section). The analysis of MNase-seq and ChIP-Seq against H3 datasets across different cell lines revealed a higher nucleosome-density signal on GC exons than AT exons (Fig. 6a, Additional file 3: Figure S5b), in agreement with a previous report showing that exons embedded in a GC-rich environment have a higher nucleosome density [11, 26]. While similar nucleosome-density signals were observed on the first exon of genes hosting either GC exons or AT exons, higher signals were observed across all internal exons of genes hosting GC exons when compared to genes hosting AT exons (Fig. 6b, c, Additional file 3: Figure S5c). In addition, a stronger signal was observed across introns of genes hosting GC exons compared to genes hosting AT exons (Fig. 6b, c), in particular across introns flanking splicing factor–activated GC exons (Fig. 6a). This could be due to the higher frequency of GCs in these introns and the lower frequency of Ts at their 3′-ends when compared to introns flanking AT exons (Fig. 2). In this setting, there was a marked nucleosome-free region both upstream and downstream of AT exons when compared to GC exons (Fig. 6a, green arrows).

The pattern of nucleosomes on GC exons and AT exons prompted us to analyze histone tail modifications that play a role in splicing regulation (see the “Introduction” section). We analyzed publicly available ChIP-seq datasets generated across different cell lines (Additional file 1: Table S1). As shown in Fig. 6d, a higher density signal corresponding to H3K4me1, H3K4me2, H3K4me3, H3K9ac, and H3K27ac was detected on GC exons when compared to AT exons. No significant differences were observed for H3K9me3, H3K27me3, H3K36me3, H3K79me3, and H4K20me1 (Fig. 6d). The pattern of histone modifications did not seem to be specific to splicing factor–regulated exons. While there was a similar signal density of histone marks on the promoter and first exon of genes hosting either AT exons or GC exons, the H3K4me3 and H3K9ac density signals were higher across all the exons of the GC exons hosting genes (Fig. 6e, f, Additional file 3: Figure S5d).

To summarize, GC and AT exons were hosted by GC- and AT-rich genes (Fig. 5a, b), respectively, that were associated with different levels of RNAPII (Fig. 5c–f) and that were differentially organized at the chromatin level (Fig. 6). Owing to the relationship between nucleotide composition bias, gene expression level, chromatin organization, and the 1D and 3D genome organization, we investigated the interplay between splicing regulation and isochores or topologically associated domains (TADs) that define the 1D or 3D human genome organization, respectively.

### GC and AT exons are hosted by genes belonging to different isochores and chromatin domains

**Fig. 7:**
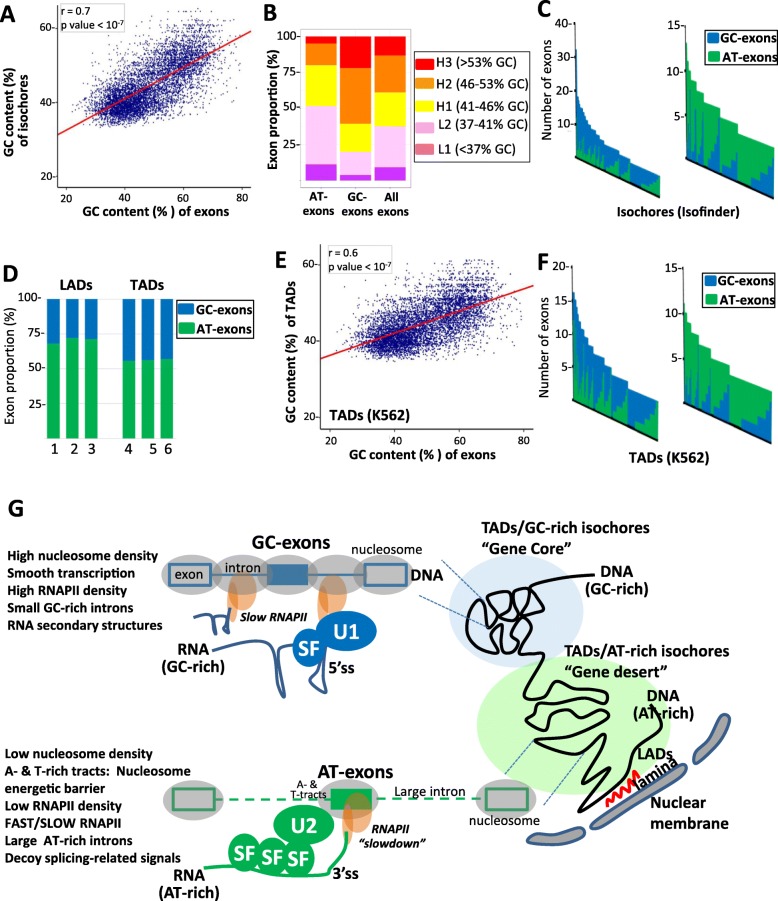
Nucleotide composition bias and genome organization. **a** Correlation between the GC content of GC exons and AT exons, and the GC content of their hosting isochores defined by ISOFINDER. **b** Proportion of AT exons, GC exons, and control exons distributed across different isochore families. **c** Number of AT exons and GC exons present in individual isochores. Only isochores containing at least five GC exons or five AT exons are represented. The left and right panels represent isochores containing preferentially GC exons or AT exons, respectively. **d** Proportion of AT exons and GC exons in LADs annotated from three different datasets (1, fibroblasts; 2, resting Jurkat cells; 3, activated Jurkat cells) and in TADs annotated from three different cell lines (4, K562; 5, IMR90; 6, MCF7). **e** Correlation between the GC content of GC exons and AT exons, and the GC content of their hosting TADs, defined in the K562 cell line. **f** Number of AT exons and GC exons present in individual TADs annotated from the K562 cell line. Only TADs containing at least five GC exons or five AT exons are represented. The left and right panels represent TADs containing preferentially GC exons or AT exons, respectively. **g** GC-rich isochores and TADs contain a large number of genes (“gene core”) that are GC-rich and contain small introns. In contrast, AT-rich isochores, TADs, and LADs contain a small number of genes (“gene desert”) that are AT-rich and contain large introns. The regional nucleotide composition bias (over dozens of kbps) increases the probability of local nucleotide composition bias (e.g., at the gene and exon levels). Local nucleotide composition bias influences local chromatin organization at the DNA level (e.g., nucleosome density and positioning) as well as the splicing process at the RNA level. The high density of nucleosomes and GC nucleotides (upper panel) could generate a “smooth” transcription across small genes, favoring synchronization between transcription and splicing. The high density of GC nucleotides increases the probability of secondary structures at the 5′ ss, with consequences on splicing recognition during the splicing process. This constraint could be alleviated by splicing factor (SF; in blue) binding to GC-rich sequences, which enhances U1 snRNP recruitment. The high density of AT nucleotide (lower panel) could favor a sharp difference between exon and intron in terms of nucleotide composition bias, which would favor nucleosome positioning on exons. A- or T-rich sequences located upstream of AT exons, as well as the presence of exonic nucleosomes, could locally (at the exon level) slow down RNAPII, favoring synchronization between transcription and splicing. The high density of AT nucleotides increases the probability of generating decoy signals, such as pseudo BPs or SF1- or U2AF2-binding sites. This constraint could be alleviated by the binding of splicing factors (SF, in green) to these decoy signals, thereby enhancing U2 snRNP recruitment

Since GC exons and AT exons are hosted by GC- and AT-rich genes, respectively (Fig. 5a, b), and since genes belong to genomic regions (or isochores) having homogenous GC content (see the “Introduction” section), we investigated whether there was a correlation between the GC content of exons and the GC content of the isochore they belong to. As shown in Fig. 7a, there was a positive correlation between the GC content of exons and the GC content of their hosting isochores. Accordingly, a larger proportion of GC exons (> 60%) than AT exons (< 25%) belongs to GC-rich isochores (> 46% of GC; Fig. 7b). Furthermore, GC exons and AT exons cluster in different isochores. Indeed, some isochores contain a larger number of GC exons than AT exons, while other isochores contain a larger number of AT exons (Fig. 7c). This result was confirmed using different annotations of isochores computed with different programs (Additional file 3: Figure S6a, b).

Some genomic domains, named lamina-associated domains (LADs), are in close proximity to the nuclear envelop [64], and some DNA domains separated by several dozens of Kbps, named topologically associated domains (TADs), are in close proximity in the nuclear space [65]. LADs and TADs have been annotated across different cell lines [64, 65]. We observed that LADs contain more frequently AT exons (~ 70%, Fig. 7d), while TADs contain a similar proportion of GC exons and AT exons (~ 55%). This is in agreement with the fact that LADs correspond to AT-rich regions [64]. Interestingly, we observed a positive correlation between the GC content of exons and the GC content of the TAD they belong to (Fig. 7e). Furthermore, GC exons and AT exons cluster in different TADs. Indeed, some TADs contain a larger number of GC than AT exons, while other TADs contain a larger number of AT exons (Fig. 7f). This result was confirmed using different annotations of TADs across different cell lines (Additional file 3: Figure S6c, d).

As already mentioned, the GC content of exons correlated with the GC content of their hosted TADs. Furthermore, the GC content of each exon correlated with the average GC content of the exons in the same TAD (Additional file 3: Figure S7a). This suggested that splicing-related features, such as the MFE at the 5′ ss, the number of splicing-related decoys, or the number of exons regulated by U1 or U2 were associated with the hosting TAD. Indeed, the values of splicing-related features were not equitably distributed among TADs and exons regulated by U1 (or U2) were more concentrated in some TADs than in others (LRT test *P* value< 10^−16^; Additional file 3: Figure S7b).

Collectively, these observations support a model where nucleotide composition bias establishes a link between genomic organization (e.g., isochores or TADs) and the splicing process.

## Discussion

The rules that govern exon recognition during splicing and that explain the dependency of some exons on different classes of splicing factors remain to be clarified. We propose that nucleotide composition bias (e.g., GC content) over large genomic regions, which plays an important role in genome organization, leaves a footprint locally at the exon level and induces constraints during the exon recognition process that can be alleviated by local chromatin organization and different classes of RNA binding proteins.

The human genome is divided in isochores corresponding to regions of varying lengths (up to several dozens of kbps) having a uniform GC content that differs from adjacent regions [19–21]. GC-rich isochores have a higher density of genes than AT-rich isochores, and GC- and gene-rich genomic regions are highly expressed [19–21, 23, 24, 61–63] (Fig. 7g). Increasing evidence indicates that the one-dimensional genome organization, defined by regional nucleotide composition bias, is related to the three-dimensional genome architecture. For example, an overlap between isochores and TADs has been reported [19, 66]. Both the relationship between the 1D and 3D genome organization and the higher density of actively transcribed genes in GC-rich regions could be explained by the physicochemical properties of GC and AT nucleotides. For example, the nature of the base stacking interactions between GC nucleotides increases DNA structural polymorphism that in turn increases DNA bendability and flexibility with consequences on DNA folding [14–16, 67–70]. Furthermore, the higher stability of G:C base pairing and frequency of GC-associated polymorphic structures have been proposed to increase the resistance of the DNA polymer to transcription-associated physical constraints. For example, the GC content is associated with the transition from the B- to Z-DNA form, the former “absorbing” the topological and torsional stresses that are generated during transcription [67–71]. In this setting, we observed that GC exons and their hosting GC-rich genes have a higher RNAPII density than AT exons and their hosting AT-rich genes (Fig. 5). Based on these observations, transcription and the three-dimensional genome organization may constrain the nucleotide composition of genomic regions, which in turn has “local” consequences on exon recognition during co-transcriptional splicing.

Supporting this possibility, first we observed a positive correlation between the GC content of exons, their flanking introns, and their hosting genes, isochores, and TADs (Figs. 1b, 5a, and 7a, e), in agreement with the notion that the GC content is uniform and homogenous regardless of the genomic scale [19–21]. Second, differential GC content is associated with specific constraints on the exon recognition process. For example, our analyses and reports from the literature support a model where a high local GC content favors the formation of RNA secondary structures that can hinder the recognition of the 5′ ss by occluding them, therefore limiting the access of U1 snRNA to the 5′ ss [3–5]. Accordingly, exons sensitive to the depletion of SNRPC and SNRNP70, two components of the U1 snRNP, as well as exons sensitive to the DDX5 and DDX17 helicases that enhance the recognition of structured 5′ ss owing to their RNA helicase activity [3–5, 55], are embedded in a GC-rich environment (Fig. 3a, b). In this setting, splicing factors that activate GC exons (for instance, hnRNPF, hnRNPH, PCBP1, RBFOX2, RBM22, RBM25, RBMX, SRSF1, SRSF5, SRSF6, and SRSF9) bind to G-, C-, or GC-rich motifs [8–10] (Additional file 3: Figure S8). Furthermore, hnRNPF, hnRNPH, RBFOX2, RBM22, RBM25, and several SRSF splicing factors are known to enhance U1 snRNP recruitment [72–76]. Therefore, a high GC content could increase the probability of generating secondary structure at the 5′ ss, which decreases exon recognition. Simultaneously, high GC content could increase the recruitment of splicing factor binding to GC-rich motifs, thereby enhancing U1 snRNP recruitment (Fig. 7g). While RNA secondary structures at the 5′ ss negatively impact exon recognition, structures at the 3′ ss favor exon recognition and replace the requirement for U2AF2 in splicing [6]. In addition, a high GC content, as well as G- and C-rich motifs upstream of the BP, enhances U2 snRNA binding and BP recognition [47, 48, 77]. Accordingly, exons embedded in a GC-rich environment are more sensitive to factors associated with U1 snRNP than those with U2 snRNP (Fig. 3g).

Our results also support a model in which a high content of AT nucleotides in large introns can negatively influence exon recognition. Indeed, high AT content upstream of exons associated with a larger number of potential BPs (Fig. 2e), in agreement with a previous report [49]. A high AT content upstream of exons also associated with a larger number of SF1- and U2AF2-binding sites (Fig. 2g, h). In this setting, increasing evidence indicates that binding of spliceosome-associated factors (e.g., SF1 or U2AF2) to pseudo-signals or decoy signals can inhibit splicing by decreasing the efficiency of spliceosome assembly [48, 53, 56–60]. These observations suggest that splicing factors that activate AT exons enhance exon recognition either by enhancing U2 snRNP recruitment or by binding to decoy splicing signals. Accordingly, splicing factors activating AT exons, including hnRNPA1, hnRNPM, RBM15, RBM39, SFPQ, and TRA2, interact with and enhance the recruitment of SF1, U2AF2, U2AF1, and/or U2 snRNP [78–83]. In addition, some of these splicing factors can compete with U2AF2 or SF1 for binding to intronic 3′-end splicing signals, and binding of splicing factors such as hnRNPA1 and PTBP1 to decoy splicing signals has been proposed to “fill up” a surplus of splicing signals and consequently enhancing the recognition of bona fide splicing sites [48, 53, 56–60]. Therefore, a high AT content could increase the probability of generating decoy splicing signals at intronic 3′-ends, which would decrease exon recognition. However, a high AT content could simultaneously increase the probability of recruiting splicing factors at decoy signals, thereby strengthening the recruitment of spliceosome-related components (e.g., SF1 and U2AF2) to bona fide splicing signals and ultimately enhancing exon recognition (Fig. 7g).

While this work focused on exons that are activated by one of the 33 analyzed splicing factors, the U1 and U2 dependency relying on exon GC content is likely a general feature. Indeed, exons (labeled oGC or oAT exons) that shared the same GC composition and flanking intron size with GC or AT exons (but that were not activated by one of the analyzed auxiliary splicing factors) had the same splicing-related features than GC or AT exons, respectively, in terms of secondary structures at 5′ss, number of decoy sites, and U1 or U2 dependency (Additional file 3: Figure S9a-e). Of note, several splicing factors activating GC or AT exons were more frequently detected by ClIP-dataset analyses in the vicinity of oGC or oAT exons, respectively (Additional file 3: Figure S9f). Obviously, oGC and oAT exons might also be activated by different sets of splicing factors. Conversely, some exons annotated as being regulated by one of the analyzed splicing factor might be indirect targets, even though ClIP-dataset analyses revealed an enrichment of the analyzed splicing factors on or in the vicinity of their activated exons (Additional file 3: Figure S9 g) and even though exons that are co-regulated by a given splicing factor share many features, as shown throughout this work.

Finally, we grouped exons regulated by different splicing factors into two main categories. However, each set of exons that are co-regulated by a given splicing factor had a different combination of properties (e.g., splicing site strength, size of exons or flanking introns, Additional file 3: Figure S1, S4, and S10). A detailed annotation of these combinations and their comparison should improve the prediction of splicing factor–regulated exons. Actually, some splicing-related features of sets of co-regulated exons could be linked to the fact that each of these sets had a specific combination of nucleotide composition bias. For example, some sets of co-regulated exons were more enriched in Gs than in Cs (e.g., SRFS2 vs SRSF3, Additional file 3: Figure S2 and S10a). We also analyzed exonic purine- or pyrimidine-biases and observed that purine- and pyrimidine-rich exons had similar properties than AT- and GC-rich exons, respectively (Additional file 3: Figure S11 and S12). Obviously, more work is needed to better understand the interdependency between splicing-related features and nucleotide composition bias both at the exon and gene levels, which can have consequences on the coupling between transcription and splicing as detailed below.

The synchronization between transcription and splicing plays a major role in the exon recognition process [11, 12, 84, 85]. We propose that the coupling between transcription and splicing operates through different mechanisms depending on the gene nucleotide composition bias, which impacts chromatin organization and RNAPII dynamics. Indeed, at the chromatin level, nucleosomes are better positioned on exons in an AT-rich context than in a GC-rich context, and there is a higher density of nucleosomes in both GC-rich exons and introns (Fig. 6a–c), as already reported [14–17]. This feature could result from the fact that exons embedded in an AT-rich context have a much higher GC content than their flanking intronic sequences, in contrast to exons embedded in a GC-rich context [11, 26] (Fig. 2a). In this setting, GC-rich stretches favor DNA wrapping around nucleosomes, because the stacking interactions between GC nucleotides allow DNA structural polymorphism that in turn increases DNA bendability; in contrast, T- and A-rich stretches form more rigid structures that create nucleosome energetic barriers [14–17]. Therefore, increasing the intronic GC content increases the probability of nucleosomes sliding from exons to introns, while increasing the density of Ts and As in exonic flanking regions creates nucleosome energetic barriers. Consequently, transcription and splicing synchronization in an AT-rich environment could depend on nucleosomes being well-positioned on exons, as these would locally slow down RNAPII and thereby favor recruitment of splicing-related factors (Fig. 7g), as previously proposed [11]. Of note, components associated with the U2 snRNP interact with chromatin-associated factors [86, 87].

Synchronization between transcription and splicing could rely on other mechanisms when exons are within a GC-rich environment. Indeed, both the higher density of nucleosomes across introns of GC-rich genes (Fig. 6a–c), and the higher stability of G:C base pairing, create constraints that reduce the velocity of RNAPII across both exons and introns. Accordingly, the rate of elongation by RNAPII is negatively correlated with gene GC content [88]. Therefore, high GC content may facilitate the synchronization between transcription and splicing by “smoothing” RNAPII dynamics all along GC-rich genes. Of note, extensive interactions between the U1 snRNP and RNAPII-associated complexes have been reported [89]; therefore, a slower speed of RNAPII across GC-rich genes may facilitate U1 snRNP recruitment (Fig. 7g). Further supporting that gene GC content plays an important role in the interplay between gene expression levels and splicing, intron removal occurs more efficiently in highly expressed genes [90], and GC-rich genomic regions associate with nuclear speckles [91, 92]. It must also be underlined that introns flanking GC-rich exons have been shown to be efficiently and probably co-transcriptionally spliced [93].

Altogether, these observations suggest a link between nucleotide composition bias, genome organization, and RNA processing (Fig. 7g). We propose a model in which transcription and genome organization constrain the nucleotide composition of DNA over dozens of kbps. In turn, nucleotide composition bias induces local (at the exon level) constraints on the splicing process by affecting specific splicing-related features. However, constraints induced by nucleotide composition bias can be alleviated by specific mechanisms. For example, although AT exons are weakened in terms of intron 3′-end definition, this can be alleviated by an interplay between exon-positioned nucleosomes, U2 snRNP–associated factors, and splicing factors that bind decoy signals and/or enhance U2 snRNP recruitment. Likewise, while GC exons are weakened at their 5′ ss because of the formation of RNA secondary structures, this can be alleviated by an interplay between a slow RNAPII, U1 snRNP–associated factors and splicing factors that bind to GC-rich sequences and enhance the recruitment of the U1 snRNP. In this model, splicing factors would enhance the recognition of exons by counteracting splicing-associated constraints resulting from nucleotide composition bias and providing room for regulatory processes such as alternative splicing.

## Materials and methods

### RNA-seq dataset analyses and establishment of GC and AT exon sets

Publicly available RNA-seq datasets generated from different cell lines transfected with siRNAs or shRNAs targeting specific splicing factors, or transfected with splicing factor expression vectors, were recovered from GEO and ENCODE (Additional file 1: Table S1). RNA-seq datasets were analyzed using FARLINE, a computational program dedicated to analyze and quantify alternative splicing variations, as previously reported [44]. This study focused on exons whose inclusion depends on at least one splicing factor. For this, the sets of exons that are activated by each analyzed splicing factor in at least one sample were defined (Additional file 2: Table S2). Exons that are regulated in an opposite way by the same splicing factor in different samples were eliminated. GC exons (or AT exons) were defined as exons whose inclusion depends on splicing factors activating exons with a median GC content higher (or lower, respectively) than control exons (threshold 49.3%) and having a median size of their smallest flanking intron smaller (or larger, respectively) than that of control exons (threshold 691 bp). Based on the results shown in Fig. 1d, GC exons were defined as exons being activated by SRSF9, PCBP1, RBMX, hnRNPF, RBFOX2, SRSF5, hnRNPH1, RBM22, RBM25, MBNL2, and SRSF6, while AT exons were defined as exons activated by TRA2A/B, RBM15, RBM39, hnRNPA2B1, KHSRP, hnRNPM, SRSF7, SFPQ, MBNL1, DAZAP1, PTBP1, hnRNPL, hnRNPK, FUS, QKI, hnRNPA1, and PCBP2. The set of control (CTRL) exons corresponds to human constitutive coding exons annotated in FASTERDB, excluding the exons activated by at least one of the analyzed splicing factors. For further analyses, exons found in the two-exon sets (i.e., exons regulated by two splicing factors of different classes) were eliminated (about 25%), leading to one list of 3182 GC exons and another of 4045 AT exons (Additional file 2: Table S2). U1 exons were defined as exons activated by the SNRPC, SNRNP70, and/or DDX5/17 factors, and U2 exons were defined as exons activated by the U2AF2, SF3B4, SF1, and/or SF3A3 factors. GA exons and CT exons were defined based on their GA and CT composition compared to the GA and CT composition of CTRL exons. All genomic annotations were from FasterDB [94].

### Heatmaps and frequency maps

Heatmaps represent the median value for a given feature of a set of splicing factor–activated exons as compared to the median value obtained for control exons. Formula (1) was used to compute the relative value of a feature D in a set of *n* exons S compared to a set of *m* control exons *C* (CTRL exons) that correspond to human constitutive coding exons annotated in FASTERDB, excluding exons regulated by at least one of the 33 analyzed splicing factors (see above).

1 $$ RFreq(D)=\frac{\mathrm{Median}\left({D}_S\right)-\mathrm{Median}\left({D}_C\right)}{\mathrm{Median}\left({D}_C\right)}\times 100 $$

where *D*_*S*_ and *D*_*C*_ are the vectors of *D* values for the sets *S* and *C.* Heatmaps of Fig. 2 b and c were generated using a *Mean* function in formula (1). A linear model (with R lm() and summary() functions) was used to compare the GC content of exons activated by each splicing factor to the GC content of the control exons. With this model, a Student’s test was computed between control exons and each set of exons activated by a splicing factor. A generalized linear model for the Poisson distribution (with glm() and summary() functions) was used to compare the counts of As, Gs, Cs, or Ts, in the 25 first nucleotides upstream exons between exons activated by each splicing factor to control exons. With this model, a Wald’s test was computed between each splicing factors activated set of exons and control exons. The sequences corresponding to the intron-exon junctions (the last 100 nucleotides of the upstream intron and the first 50 nucleotides the exon) were recovered for each exon. The mean frequency of a given nucleotide was computed at each window position using sliding window with a size and a step of 20 and 1 nucleotide, respectively. The same procedure was applied for the sequences corresponding to exon-intron junctions defined as the last 50 nucleotides of the exon and the first 100 nucleotides of the downstream intron.

### Splice site scores, minimum free energy, BP predictions, U2 binding energy, and motif count

The splice site scores were calculated for each FasterDB exons with MaxEntScan (http://genes.mit.edu/burgelab/maxent/Xmaxentscan_scoreseq.html). The minimum free energy (MFE) was computed from exon-intron junction sequences (25 nucleotides within the intron and 25 nucleotides within the exon) using RNAFold from the ViennaRNA package [46] (v2.4.1; http://rna.tbi.univie.ac.at/cgi-bin/RNAWebSuite/RNAfold.cgi). These sequences were split in two groups: the sequences centered on the 5′ ss and the sequences centered on 3′ ss. The Anscombe transformation was applied on MFE values to obtain Gaussian distributed values. An ANOVA model (with R, aov() function) was built and statistical differences between every couple of group of exons was tested with a Tukey’s test. Differences between MFE of exons activated by each spliceosome-associated factor and control exons were tested using a linear model (with R lm() and summary() functions). The number of branch points in a given sequence corresponding to the 100 nucleotides preceding 3′ ss was computed with SVM-BP finder (http://regulatorygenomics.upf.edu/Software/SVM_BP/). Only branch point sites with a svm score > 0 were considered. The U2 snRNA binding energy corresponds to the number of hydrogen bounds between the nucleotides surrounding the branch point of an RNA sequence (without the branch point adenine) and the branch point binding sequence of U2 snRNA. The RNAduplex script in the ViennaRNA package (v2.4.1) was used to determine the optimal hybridization structure between the branch point binding sequence of U2 snRNA (GUGUAGUA) and the RNA sequence. The RNA sequence is composed of 5 nucleotides before and 3 after the branch point. Then, the sum of hydrogen bounds forming between the RNA and the U2 sequence was computed. The number of TNA motifs was computed in the last 50 nucleotides of each intron. To test the differences for the three features mentioned above between groups of exons activated by spliceosome-associated factors and the control group of exons, the same procedure as the one applied for Fig. 2 b and c was used (see previous section) (Fig. 3c, d). To test the differences between every couple of group of exons, a Tukey’s test was used (with R, glh function (library multcomp)) (Fig. 2e, g, left panel). T-rich low-complexity sequences were computed between the 75th to the 35th nucleotides upstream the 3′ ss, using a sliding window (size 4, step 1 and at least three Ts). Statistical differences were tested by using a linear model for the negative binomial distribution (with glm.nb() function, library MASS). Statistical differences between every couple of group of exons were tested using a Tukey’s test (R, glh function).

#### *V* value: exons regulation by U1 or U2 snRNP–associated factors

Difference between the proportion of GC exons and AT exons depending on spliceosome-associated factors was tested by a randomization procedure. For each spliceosome-associated factor, 10,000 subsamples of AT exons (with the size of the GC-exons set) were generated and the proportion of exons activated by the factor for each sample was computed. The empirical *P* value *P*_emp_ was computed as:

$$ {P}_{\mathrm{emp}}=\max \left(\frac{\min \left(k,l\right)}{\mathrm{10,000}},{10}^{-4}\right) $$

with *k* the number of AT-exon samples with a higher or equal proportion of exons activated by the factor of interest as compared to GC exons and *l* the number of AT-exons samples with a lower or equal proportion of exons activated by the factor of interest as compared to GC exons.

The *V* value for each spliceosome-associated factor was computed using the formula:

$$ v=\log 10\left({P}_{\mathrm{emp}}\right)\times s $$

where *s* = 1 if *k* > *l*; *s* = − 1 otherwise.

### Statistical analysis at gene level

To test whether the GC content of genes hosting AT exons (AT genes) or GC exons (GC genes) was different, the GC content of the genes according to their size and their group was modeled with an ANOVA model (in R, aov() function). A Tukey’s test on this model was computed to compare between all the possible couples of gene groups (AT, GC, and control genes). To test if the median intron size was different for each couple of AT, GC, and control groups of genes, a Wilcoxon’s test was performed. To test if the gene size of GC, AT, and control genes was different, we built an ANOVA model (in R, aov() function). Then, a Tukey’s test was performed on this model. For those analyses, genes hosting both AT and GC exons were not considered.

### CLIP-seq dataset analyses

Bed files from publicly available CLIP-seq datasets generated using U2AF2 antibodies (GSE83923, GSM2221657; GSE61603 or GSM1509288) were used to generate density maps. The bed files were first sorted and transformed into bedGraph files using the bedtools suite (v2.25.0). The bedGraph files were then converted into bigWig files using bedGraphToBigWig (v4). The 5′ ss and 3′ ss regions (comprising the ss, 200 nucleotides into the intron and 50 nucleotides into the exon) were considered. The proportions of GC or AT exons, or exons activated by at least one U1-associated factor (e.g., SNRPC, DDX5/17, and SNRNP70) or at least one U2-associated factor (e.g., U2AF2, SF3B4, SF1, and SF3A3) with CLIP peak signals at each nucleotide position of the 5′ ss and 3′ ss regions, were computed.

### Analysis of MNase datasets and RNAPII, H3, and histone mark–related ChIP-seq datasets

ChIP-seq or MNase-seq datasets were recovered from Cistrome, ENCODE, and GEO databases (Additional file 1: Table S1). Coverage files (BigWig) were directly downloaded from Cistrome and RNAPII ChIP-seq from ENCODE. Annotations were lifted over from hg38 to hg19 if the coverage file came from Cistrome database. Otherwise, raw data were downloaded for analysis with homemade pipelines. Reads were trimmed and filtered for a minimum length of 25b using Cutadapt 1.16 (options: -m 25), trimmed at their 3′ end for a minimum quality of 20 (-q 20) and then filtered for minimum length of 25b (–m 25). The processed reads were mapped to hg19 with Bowtie2 2.3.3 (options: --very-sensitive --fr -I 100 -X 300 --no-mixed) and filtered for mapping quality over 10 with samtools view 1.6 (options: -b -q 10). For ChIP-seq experiments generated using sonication only, duplicates were removed with homemade tools, which check for coordinates and CIGAR of the Read and the Read 2 adaptor sequence if paired-end sequencing was used. Fragments were reconstituted from the reads, and fragment-coverage files were built using MACS2 2.1.1.20160309 (options: -g hs -B). The metaplots of ChIP/MNase-seq on genes were generated by recovering the fragment coverage (promoter – 1500b/+ 500 from the TSS; first exon, internal exons and introns: according to the coordinates of the annotation; splicing factor–regulated exons − 100/+ 100 from the center, or 500b into the intron and 50b into the exon from the splicing site [MNase and H3]; whole gene: according to the coordinates of the annotations and − 200/+ 200 from the annotation). In the case of RNAPII coverage, only exons regulated in the corresponding cell line, or annotation from their hosting gene, were considered. For internal exons and introns, the coverages of the annotations from the same gene were concatenated respecting their genomic order. The coverages recovered according to the coordinates of the annotations were the split into 1000 bins. The first 199 bins of “internal exons” or of introns were removed to avoid displaying signals influenced by the promoter. Metaplots were built by computing, at each position or bin, the average coverage across the annotations. Statistics were done by comparing average coverage in the annotations from two groups, which were cell-line specific, with a Wilcoxon’s test. In Fig. 5d, the average of the mean coverage on each regulated exons (− 100/+ 100 from center) was computed. For each ChIP-seq experiment, the ratio of the averages (“GC” - “AT”/max(“GC”, “AT”)) was computed and used to build the boxplot in Fig. 6c, and the statistics were computed with a Wilcoxon’s test of the average per experiment of GC compared to AT exons.

### Isochores, LADs, TADs, and CLIP-seq dataset analyses

Chromosome coordinates of isochores, LADs, and TADs were recovered from previous publications or GEO (see Additional file 4: Table S3). The bedtool intersect command was used to determine the isochore and TAD and LAD regions to which each exon belongs. The percentage of GC, and the number of exons (GC- or AT exons), present in each annotated isochore, LAD, or TAD was calculated (Additional file 4: Table S3).

### Statistical analysis at TAD level

After labelling exons accordingly to their hosting TADs (see above and Additional file 4: Table S3), we tested whether different splicing-related features were equally distributed between TADs. We modeled different features by TADs as a mixed effect on the number of exons with more than two branch points, or the number of U1 and U2 exons, while accounting for the size of TADs (i.e., the number of hosted exons). The models were implemented using the glmer function (package lme4 [1]) with family = binomial in R software (see Additional file 3: Figure S7). A likelihood ratio test (R software, function anova with test = “Chisq”) was used to test the effect of TADs. The same procedure was used with the function glmer.nb (package lme4) in R to model the number in TADs of TNA motifs and T-rich low-complexity sequences. The same procedure was used with the function lmer (package lme4) in R to model MFE values in TADs.

### Cell culture, transfections, RNA preparation, and RT-(q)PCR

The human MCF-7 and HeLa cell lines were cultured in DMEM medium (GIBCO) complemented with 10% FBS, 1% glutamine, and 1% penicillin/streptomycin. Cells were reverse-transfected using Lipofectamine RNAiMax (Thermo Fisher) following the manufacturer’s instructions. Thirty nanomolars of siRNA was mixed with 100 nM of 2′-O-methylated antisense RNA oligonucleotides (AON) targeting intronic sequences. Cells were harvested 48 h after transfection, and total RNA was isolated using TriReagent. 1.5 μg of total RNA was DNAse-treated and retro-transcribed using the Maxima First Strand cDNA Kit (ThermoFisher) following the manufacturer’s instructions. RT-PCR reactions were performed using 12.5 ng cDNA and 0.5 U GoTaq® DNA polymerase (Promega). qRT-PCR reactions were performed using 7.5 ng cDNA and the SYBR® Premix Ex Taq (Takara). Melting curves were controlled to rule out the existence of non-specific products. Relative DNA levels were calculated using the ΔΔCt method (using the average Ct obtained from technical duplicates or triplicates). siRNA, AON, and primer sequences are provided in Additional file 1: Table S1. The cell lines were authenticated by ATCC.

## Supplementary information

**Additional file 1: Table S1.** GEO number of publicly available large-scale datasets analyzed in this work and siRNA-, AON-, and primer-sequences.

**Additional file 2: Table S2.** List of exons activated by each analyzed splicing factors and lists of the GC-exons and AT-exons.

**Additional file 3: Figure S1.** Violin plots representing the relative splicing site scores. **Figure S2.** Violin plots representing the relative adenine, cytosine, guanine, and thymine frequencies. **Figure S3.** GC- and AT-nucleotide composition bias. **Figure S4.** Intron and exon size. **Figure S5.** ChIP-seq and MNase-seq analyses. **Figure S6.** Isochore, TAD and LAD analyses. **Figure S7.** Statistical models. **Figure S8.** Splicing factor binding motifs. **Figure S9.** Analyses of oGC-exons or oAT-exons. **Figure S10.** Analyses of SRSF2-, SRSF3, and hnRNPC-regulated exons. **Figures S11 and S12.** Analyses of GA and CT-exons.

**Additional file 4 Table S3.** Annotation of isochores, LADs, and TADs.

**Additional file 5.** Review history.

## Data Availability

Accession numbers of published ChIP-seq, RNA-seq, MNase-seq, and CLIP-seq datasets are included in Additional file 1: Table S1, and the data related to Isochores, LADs, and TADs are included in Additional file 4: Table S3.

## References

[1] Wahl MC, Will CL, Luhrmann R (2009). The spliceosome: design principles of a dynamic RNP machine. Cell.

[2] Piao M, Sun L, Zhang QC (2017). RNA regulations and functions decoded by transcriptome-wide RNA structure probing. Genomics Proteomics Bioinformatics.

[3] Zhang J, Kuo CC, Chen L (2011). GC content around splice sites affects splicing through pre-mRNA secondary structures. BMC Genomics.

[4] Kar A, Fushimi K, Zhou X, Ray P, Shi C, Chen X, Liu Z, Chen S, Wu JY (2011). RNA helicase p68 (DDX5) regulates tau exon 10 splicing by modulating a stem-loop structure at the 5′ splice site. Mol Cell Biol.

[5] Lambert MP, Terrone S, Giraud G, Benoit-Pilven C, Cluet D, Combaret V, Mortreux F, Auboeuf D, Bourgeois CF (2018). The RNA helicase DDX17 controls the transcriptional activity of REST and the expression of proneural microRNAs in neuronal differentiation. Nucleic Acids Res.

[6] Lin CL, Taggart AJ, Lim KH, Cygan KJ, Ferraris L, Creton R, Huang YT, Fairbrother WG (2016). RNA structure replaces the need for U2AF2 in splicing. Genome Res.

[7] Fu XD, Ares M (2014). Context-dependent control of alternative splicing by RNA-binding proteins. Nat Rev Genet.

[8] Dominguez D, Freese P, Alexis MS, Su A, Hochman M, Palden T, Bazile C, Lambert NJ, Van Nostrand EL, Pratt GA (2018). Sequence, structure, and context preferences of human RNA binding proteins. Mol Cell.

[9] Giudice G, Sanchez-Cabo F, Torroja C, Lara-Pezzi E. ATtRACT-a database of RNA-binding proteins and associated motifs. Database (Oxford). 2016;1–9. 10.1093/database/baw035 PMC4823821 27055826

[10] Ray D, Kazan H, Cook KB, Weirauch MT, Najafabadi HS, Li X, Gueroussov S, Albu M, Zheng H, Yang A (2013). A compendium of RNA-binding motifs for decoding gene regulation. Nature.

[11] Hollander D, Naftelberg S, Lev-Maor G, Kornblihtt AR, Ast G (2016). How are short exons flanked by long introns defined and committed to splicing?. Trends Genet.

[12] Naftelberg S, Schor IE, Ast G, Kornblihtt AR (2015). Regulation of alternative splicing through coupling with transcription and chromatin structure. Annu Rev Biochem.

[13] Dujardin G, Lafaille C, de la Mata M, Marasco LE, Munoz MJ, Le Jossic-Corcos C, Corcos L, Kornblihtt AR (2014). How slow RNA polymerase II elongation favors alternative exon skipping. Mol Cell.

[14] Iyer VR (2012). Nucleosome positioning: bringing order to the eukaryotic genome. Trends Cell Biol.

[15] Tilgner H, Nikolaou C, Althammer S, Sammeth M, Beato M, Valcarcel J, Guigo R (2009). Nucleosome positioning as a determinant of exon recognition. Nat Struct Mol Biol.

[16] Segal E, Widom J (2009). Poly(dA:dT) tracts: major determinants of nucleosome organization. Curr Opin Struct Biol.

[17] Schwartz S, Meshorer E, Ast G (2009). Chromatin organization marks exon-intron structure. Nat Struct Mol Biol.

[18] Luco RF, Pan Q, Tominaga K, Blencowe BJ, Pereira-Smith OM, Misteli T (2010). Regulation of alternative splicing by histone modifications. Science.

[19] Jabbari K, Bernardi G (2017). An isochore framework underlies chromatin architecture. PLoS One.

[20] Oliver JL, Carpena P, Hackenberg M, Bernaola-Galvan P (2004). IsoFinder: computational prediction of isochores in genome sequences. Nucleic Acids Res.

[21] Costantini M, Clay O, Auletta F, Bernardi G (2006). An isochore map of human chromosomes. Genome Res.

[22] Costantini M, Musto H (2017). The isochores as a fundamental level of genome structure and organization: a general overview. J Mol Evol.

[23] Arhondakis S, Auletta F, Bernardi G (2011). Isochores and the regulation of gene expression in the human genome. Genome Biol Evol.

[24] Versteeg R, van Schaik BD, van Batenburg MF, Roos M, Monajemi R, Caron H, Bussemaker HJ, van Kampen AH (2003). The human transcriptome map reveals extremes in gene density, intron length, GC content, and repeat pattern for domains of highly and weakly expressed genes. Genome Res.

[25] Burge C, Karlin S (1997). Prediction of complete gene structures in human genomic DNA. J Mol Biol.

[26] Amit M, Donyo M, Hollander D, Goren A, Kim E, Gelfman S, Lev-Maor G, Burstein D, Schwartz S, Postolsky B (2012). Differential GC content between exons and introns establishes distinct strategies of splice-site recognition. Cell Rep.

[27] Fontrodona N, Aube F, Claude JB, Polveche H, Lemaire S, Tranchevent LC, Modolo L, Mortreux F, Bourgeois CF, Auboeuf D (2019). Interplay between coding and exonic splicing regulatory sequences. Genome Res.

[28] Consortium EP (2012). An integrated encyclopedia of DNA elements in the human genome. Nature.

[29] Choudhury R, Roy SG, Tsai YS, Tripathy A, Graves LM, Wang Z (2014). The splicing activator DAZAP1 integrates splicing control into MEK/Erk-regulated cell proliferation and migration. Nat Commun.

[30] Xu Y, Gao XD, Lee JH, Huang H, Tan H, Ahn J, Reinke LM, Peter ME, Feng Y, Gius D (2014). Cell type-restricted activity of hnRNPM promotes breast cancer metastasis via regulating alternative splicing. Genes Dev.

[31] Best A, James K, Dalgliesh C, Hong E, Kheirolahi-Kouhestani M, Curk T, Xu Y, Danilenko M, Hussain R, Keavney B (2014). Human Tra2 proteins jointly control a CHEK1 splicing switch among alternative and constitutive target exons. Nat Commun.

[32] Kim E, Ilagan JO, Liang Y, Daubner GM, Lee SC, Ramakrishnan A, Li Y, Chung YR, Micol JB, Murphy ME (2015). SRSF2 mutations contribute to myelodysplasia by mutant-specific effects on exon recognition. Cancer Cell.

[33] Misra A, Ou J, Zhu LJ, Green MR (2015). Global analysis of CPSF2-mediated alternative splicing: integration of global iCLIP and transcriptome profiling data. Genom Data.

[34] Ge Z, Quek BL, Beemon KL, Hogg JR. Polypyrimidine tract binding protein 1 protects mRNAs from recognition by the nonsense-mediated mRNA decay pathway. Elife. 2016;5:e11155. 10.7554/eLife.11155 PMC4764554 26744779

[35] Reber S, Stettler J, Filosa G, Colombo M, Jutzi D, Lenzken SC, Schweingruber C, Bruggmann R, Bachi A, Barabino SM (2016). Minor intron splicing is regulated by FUS and affected by ALS-associated FUS mutants. EMBO J.

[36] Liu N, Zhou KI, Parisien M, Dai Q, Diatchenko L, Pan T (2017). N6-methyladenosine alters RNA structure to regulate binding of a low-complexity protein. Nucleic Acids Res.

[37] Appocher C, Mohagheghi F, Cappelli S, Stuani C, Romano M, Feiguin F, Buratti E (2017). Major hnRNP proteins act as general TDP-43 functional modifiers both in Drosophila and human neuronal cells. Nucleic Acids Res.

[38] Fei T, Chen Y, Xiao T, Li W, Cato L, Zhang P, Cotter MB, Bowden M, Lis RT, Zhao SG (2017). Genome-wide CRISPR screen identifies HNRNPL as a prostate cancer dependency regulating RNA splicing. Proc Natl Acad Sci U S A.

[39] Juan-Mateu J, Alvelos MI, Turatsinze JV, Villate O, Lizarraga-Mollinedo E, Grieco FA, Marroqui L, Bugliani M, Marchetti P, Eizirik DL (2018). SRp55 regulates a splicing network that controls human pancreatic beta-cell function and survival. Diabetes.

[40] Huang H, Zhang J, Harvey SE, Hu X, Cheng C (2017). RNA G-quadruplex secondary structure promotes alternative splicing via the RNA-binding protein hnRNPF. Genes Dev.

[41] Li Y, Bakke J, Finkelstein D, Zeng H, Wu J, Chen T (2018). HNRNPH1 is required for rhabdomyosarcoma cell growth and survival. Oncogenesis.

[42] Perron G, Jandaghi P, Solanki S, Safisamghabadi M, Storoz C, Karimzadeh M, Papadakis AI, Arseneault M, Scelo G, Banks RE (2018). A general framework for interrogation of mRNA stability programs identifies RNA-binding proteins that govern Cancer Transcriptomes. Cell Rep.

[43] Huelga SC, Vu AQ, Arnold JD, Liang TY, Liu PP, Yan BY, Donohue JP, Shiue L, Hoon S, Brenner S (2012). Integrative genome-wide analysis reveals cooperative regulation of alternative splicing by hnRNP proteins. Cell Rep.

[44] Benoit-Pilven C, Marchet C, Chautard E, Lima L, Lambert MP, Sacomoto G, Rey A, Cologne A, Terrone S, Dulaurier L (2018). Complementarity of assembly-first and mapping-first approaches for alternative splicing annotation and differential analysis from RNAseq data. Sci Rep.

[45] Vinogradov AE (2001). Within-intron correlation with base composition of adjacent exons in different genomes. Gene.

[46] Gruber AR, Bernhart SH, Lorenz R (2015). The ViennaRNA web services. Methods Mol Biol.

[47] Mercer TR, Clark MB, Andersen SB, Brunck ME, Haerty W, Crawford J, Taft RJ, Nielsen LK, Dinger ME, Mattick JS (2015). Genome-wide discovery of human splicing branchpoints. Genome Res.

[48] Paggi JM, Bejerano G (2018). A sequence-based, deep learning model accurately predicts RNA splicing branchpoints. RNA.

[49] Corvelo A, Hallegger M, Smith CW, Eyras E (2010). Genome-wide association between branch point properties and alternative splicing. PLoS Comput Biol.

[50] Singh R, Valcarcel J, Green MR (1995). Distinct binding specificities and functions of higher eukaryotic polypyrimidine tract-binding proteins. Science.

[51] Rosel-Hillgartner TD, Hung LH, Khrameeva E, Le Querrec P, Gelfand MS, Bindereif A (2013). A novel intra-U1 snRNP cross-regulation mechanism: alternative splicing switch links U1C and U1-70K expression. PLoS Genet.

[52] Ilagan JO, Ramakrishnan A, Hayes B, Murphy ME, Zebari AS, Bradley P, Bradley RK (2015). U2AF1 mutations alter splice site recognition in hematological malignancies. Genome Res.

[53] Shao C, Yang B, Wu T, Huang J, Tang P, Zhou Y, Zhou J, Qiu J, Jiang L, Li H (2014). Mechanisms for U2AF to define 3′ splice sites and regulate alternative splicing in the human genome. Nat Struct Mol Biol.

[54] Kralovicova J, Knut M, Cross NC, Vorechovsky I (2015). Identification of U2AF(35)-dependent exons by RNA-Seq reveals a link between 3′ splice-site organization and activity of U2AF-related proteins. Nucleic Acids Res.

[55] Dardenne E, Polay Espinoza M, Fattet L, Germann S, Lambert MP, Neil H, Zonta E, Mortada H, Gratadou L, Deygas M (2014). RNA helicases DDX5 and DDX17 dynamically orchestrate transcription, miRNA, and splicing programs in cell differentiation. Cell Rep.

[56] Howard JM, Lin H, Wallace AJ, Kim G, Draper JM, Haeussler M, Katzman S, Toloue M, Liu Y, Sanford JR (2018). HNRNPA1 promotes recognition of splice site decoys by U2AF2 in vivo. Genome Res.

[57] Pineda JMB, Bradley RK (2018). Most human introns are recognized via multiple and tissue-specific branchpoints. Genes Dev.

[58] Sutandy FXR, Ebersberger S, Huang L, Busch A, Bach M, Kang HS, Fallmann J, Maticzka D, Backofen R, Stadler PF (2018). In vitro iCLIP-based modeling uncovers how the splicing factor U2AF2 relies on regulation by cofactors. Genome Res.

[59] Chen L, Weinmeister R, Kralovicova J, Eperon LP, Vorechovsky I, Hudson AJ, Eperon IC (2017). Stoichiometries of U2AF35, U2AF65 and U2 snRNP reveal new early spliceosome assembly pathways. Nucleic Acids Res.

[60] Wu T, Fu XD (2015). Genomic functions of U2AF in constitutive and regulated splicing. RNA Biol.

[61] Gul IS, Staal J, Hulpiau P, De Keuckelaere E, Kamm K, Deroo T, Sanders E, Staes K, Driege Y, Saeys Y (2018). GC content of early metazoan genes and its impact on gene expression levels in mammalian cell lines. Genome Biol Evol.

[62] Urrutia AO, Hurst LD (2003). The signature of selection mediated by expression on human genes. Genome Res.

[63] Kudla G, Lipinski L, Caffin F, Helwak A, Zylicz M (2006). High guanine and cytosine content increases mRNA levels in mammalian cells. PLoS Biol.

[64] van Steensel B, Belmont AS (2017). Lamina-associated domains: links with chromosome architecture, heterochromatin, and gene repression. Cell.

[65] Rowley MJ, Corces VG (2018). Organizational principles of 3D genome architecture. Nat Rev Genet.

[66] Liu S, Zhang L, Quan H, Tian H, Meng L, Yang L, Feng H, Gao YQ (2018). From 1D sequence to 3D chromatin dynamics and cellular functions: a phase separation perspective. Nucleic Acids Res.

[67] Dans PD, Faustino I, Battistini F, Zakrzewska K, Lavery R, Orozco M (2014). Unraveling the sequence-dependent polymorphic behavior of d(CpG) steps in B-DNA. Nucleic Acids Res.

[68] Reymer A, Zakrzewska K, Lavery R (2018). Sequence-dependent response of DNA to torsional stress: a potential biological regulation mechanism. Nucleic Acids Res.

[69] Vinogradov AE, Anatskaya OV (2017). DNA helix: the importance of being AT-rich. Mamm Genome.

[70] Vinogradov AE (2003). DNA helix: the importance of being GC-rich. Nucleic Acids Res.

[71] Shin So-I., Ham Seokjin, Park Jihwan, Seo Seong Hye, Lim Chae Hyun, Jeon Hyeongrin, Huh Jounghyun, Roh Tae-Young (2016). Z-DNA-forming sites identified by ChIP-Seq are associated with actively transcribed regions in the human genome. DNA Research.

[72] Wang E, Cambi F (2009). Heterogeneous nuclear ribonucleoproteins H and F regulate the proteolipid protein/DM20 ratio by recruiting U1 small nuclear ribonucleoprotein through a complex array of G runs. J Biol Chem.

[73] Huang SC, Ou AC, Park J, Yu F, Yu B, Lee A, Yang G, Zhou A, Benz EJ (2012). RBFOX2 promotes protein 4.1R exon 16 selection via U1 snRNP recruitment. Mol Cell Biol.

[74] Akker SA, Misra S, Aslam S, Morgan EL, Smith PJ, Khoo B, Chew SL (2007). Pre-spliceosomal binding of U1 small nuclear ribonucleoprotein (RNP) and heterogenous nuclear RNP E1 is associated with suppression of a growth hormone receptor pseudoexon. Mol Endocrinol.

[75] Rasche N, Dybkov O, Schmitzova J, Akyildiz B, Fabrizio P, Luhrmann R (2012). Cwc2 and its human homologue RBM22 promote an active conformation of the spliceosome catalytic centre. EMBO J.

[76] Cho S, Hoang A, Sinha R, Zhong XY, Fu XD, Krainer AR, Ghosh G (2011). Interaction between the RNA binding domains of Ser-Arg splicing factor 1 and U1-70K snRNP protein determines early spliceosome assembly. Proc Natl Acad Sci U S A.

[77] Murray JI, Voelker RB, Henscheid KL, Warf MB, Berglund JA (2008). Identification of motifs that function in the splicing of non-canonical introns. Genome Biol.

[78] Tavanez JP, Madl T, Kooshapur H, Sattler M, Valcarcel J (2012). hnRNP A1 proofreads 3′ splice site recognition by U2AF. Mol Cell.

[79] Gozani O, Patton JG, Reed R (1994). A novel set of spliceosome-associated proteins and the essential splicing factor PSF bind stably to pre-mRNA prior to catalytic step II of the splicing reaction. EMBO J.

[80] Zhang L, Tran NT, Su H, Wang R, Lu Y, Tang H, Aoyagi S, Guo A, Khodadadi-Jamayran A, Zhou D, et al. Cross-talk between PRMT1-mediated methylation and ubiquitylation on RBM15 controls RNA splicing. Elife. 2015;4:e07938. 10.7554/eLife.07938 PMC4775220 26575292

[81] Mai S, Qu X, Li P, Ma Q, Cao C, Liu X (1859). Global regulation of alternative RNA splicing by the SR-rich protein RBM39. Biochim Biophys Acta.

[82] Wu JY, Maniatis T (1993). Specific interactions between proteins implicated in splice site selection and regulated alternative splicing. Cell.

[83] Cho S, Moon H, Loh TJ, Oh HK, Cho S, Choy HE, Song WK, Chun JS, Zheng X, Shen H (1839). hnRNP M facilitates exon 7 inclusion of SMN2 pre-mRNA in spinal muscular atrophy by targeting an enhancer on exon 7. Biochim Biophys Acta.

[84] Aslanzadeh V, Huang Y, Sanguinetti G, Beggs JD (2018). Transcription rate strongly affects splicing fidelity and cotranscriptionality in budding yeast. Genome Res.

[85] Fong N, Kim H, Zhou Y, Ji X, Qiu J, Saldi T, Diener K, Jones K, Fu XD, Bentley DL (2014). Pre-mRNA splicing is facilitated by an optimal RNA polymerase II elongation rate. Genes Dev.

[86] Kfir N, Lev-Maor G, Glaich O, Alajem A, Datta A, Sze SK, Meshorer E, Ast G (2015). SF3B1 association with chromatin determines splicing outcomes. Cell Rep.

[87] Allemand E, Myers MP, Garcia-Bernardo J, Harel-Bellan A, Krainer AR, Muchardt C (2016). A broad set of chromatin factors influences splicing. PLoS Genet.

[88] Veloso A, Kirkconnell KS, Magnuson B, Biewen B, Paulsen MT, Wilson TE, Ljungman M (2014). Rate of elongation by RNA polymerase II is associated with specific gene features and epigenetic modifications. Genome Res.

[89] Chi B, O'Connell JD, Yamazaki T, Gangopadhyay J, Gygi SP, Reed R (2018). Interactome analyses revealed that the U1 snRNP machinery overlaps extensively with the RNAP II machinery and contains multiple ALS/SMA-causative proteins. Sci Rep.

[90] Pai AA, Henriques T, McCue K, Burkholder A, Adelman K, Burge CB. The kinetics of pre-mRNA splicing in the Drosophila genome and the influence of gene architecture. Elife. 2017;6:e32537. 10.7554/eLife.32537 PMC5762160 29280736

[91] Smith KP, Moen PT, Wydner KL, Coleman JR, Lawrence JB (1999). Processing of endogenous pre-mRNAs in association with SC-35 domains is gene specific. J Cell Biol.

[92] Shopland LS, Johnson CV, Byron M, McNeil J, Lawrence JB (2003). Clustering of multiple specific genes and gene-rich R-bands around SC-35 domains: evidence for local euchromatic neighborhoods. J Cell Biol.

[93] Tilgner H, Knowles DG, Johnson R, Davis CA, Chakrabortty S, Djebali S, Curado J, Snyder M, Gingeras TR, Guigo R (2012). Deep sequencing of subcellular RNA fractions shows splicing to be predominantly co-transcriptional in the human genome but inefficient for lncRNAs. Genome Res.

[94] Mallinjoud P, Villemin JP, Mortada H, Polay Espinoza M, Desmet FO, Samaan S, Chautard E, Tranchevent LC, Auboeuf D (2014). Endothelial, epithelial, and fibroblast cells exhibit specific splicing programs independently of their tissue of origin. Genome Res.

