# Supplementary material for: Characterizing the interplay between gene nucleotide composition bias and splicing
Source: Genome Biol. 2019 Nov 29;20:259. doi: 10.1186/s13059-019-1869-y (PMC6883713; doi:10.1186/s13059-019-1869-y)
Supplement: Supplementary file 3 — Additional file 3: Figure S1. Violin plots representing the relative splicing site scores. Figure S2. Violin plots representing the relative adenine, cytosine, guanine, and thymine frequencies. Figure S3. GC- and AT-nucleotide composition bias. Figure S4. Intron and exon size. Figure S5. ChIP-seq and MNase-seq analyses. Figure S6. Isochore, TAD and LAD analyses. Figure S7. Statistical models. Figure S8. Splicing factor binding motifs. Figure S9. Analyses of oGC-exons or oAT-exons. Figure S10. Analyses of SRSF2-, SRSF3, and hnRNPC-regulated exons. Figures S11 and S12. Analyses of GA and CT-exons. [file 13059_2019_1869_MOESM3_ESM.pdf]

Fig S1

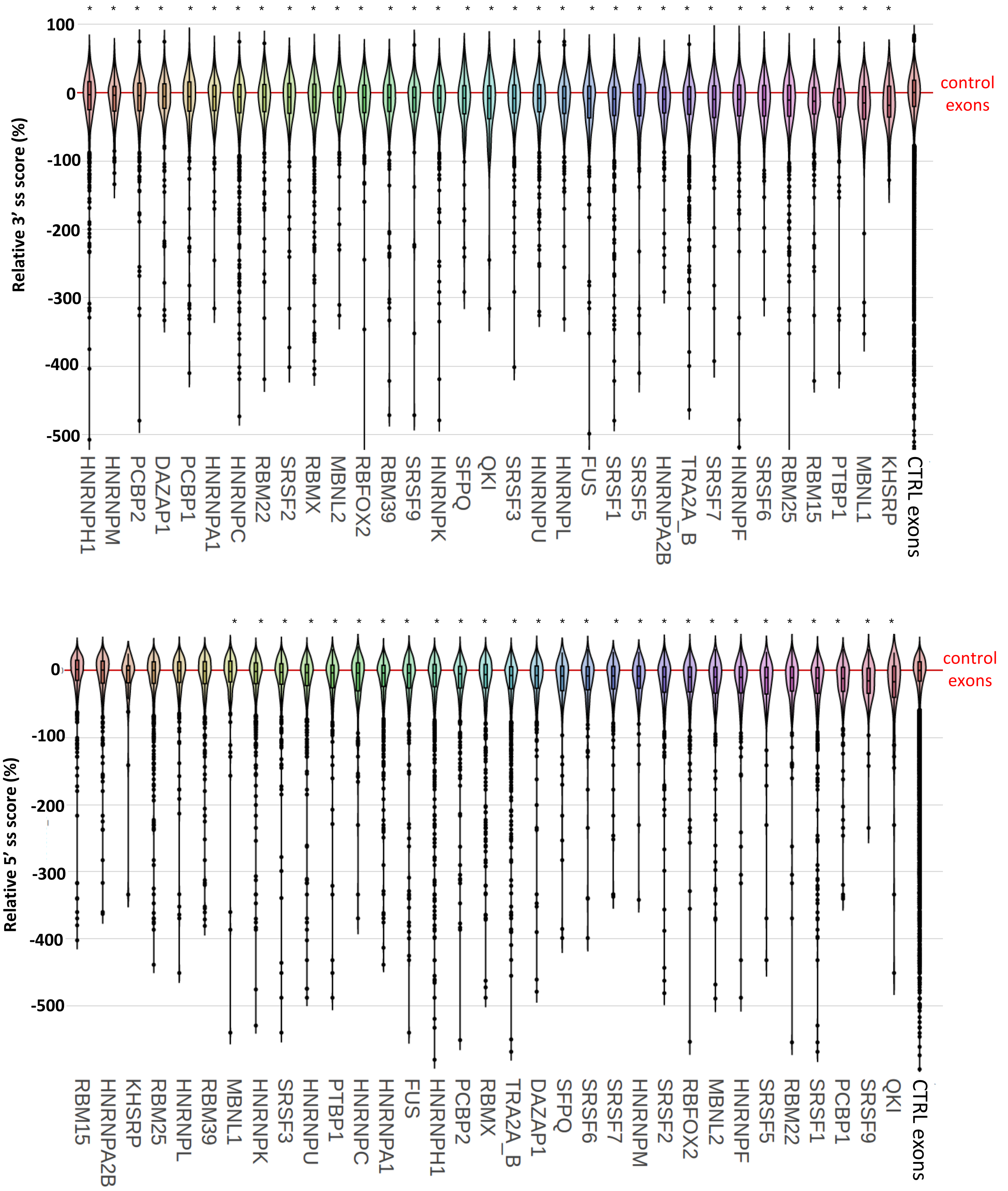

Fig S1: Violin plots representing the relative 3’ ss score (upper panel) and the relative 5’ ss score (lower panel) for each set of splicing-factor activated exons, when compared to control exons. CCE=control coding exons; (*) Wilcoxon’s test FDR < 0.05.

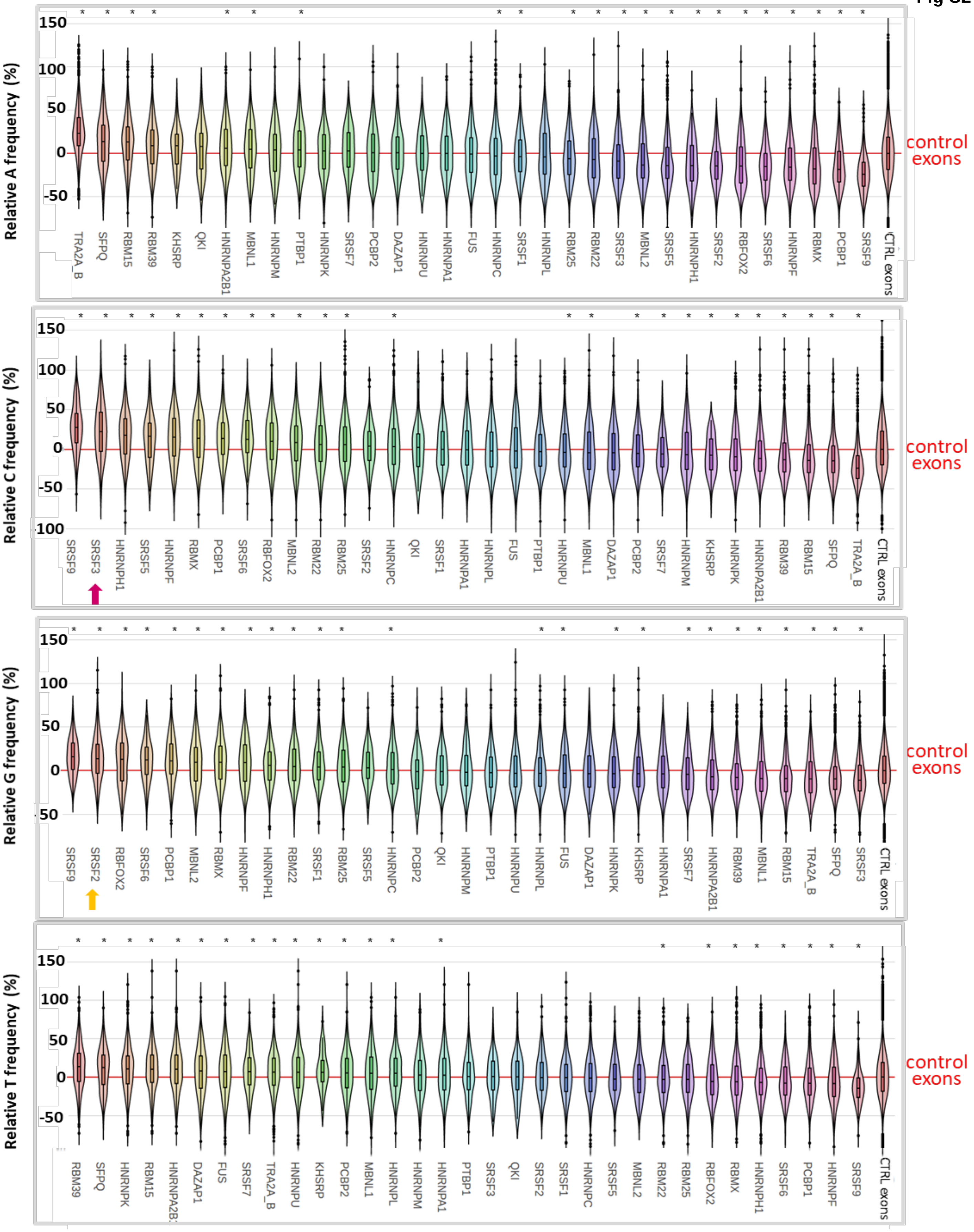

Fig S2: Violin plots representing the relative adenine, cytosine, guanine, and thymine frequencies for each set of splicing-factor activated exons, when compared to control exons (CTRL exons); (*) Student's test FDR < 0.05. SRSF2- and SRSF3-activated exons are differentially enriched in G- and C-nucleotides (see also Fig. S10).

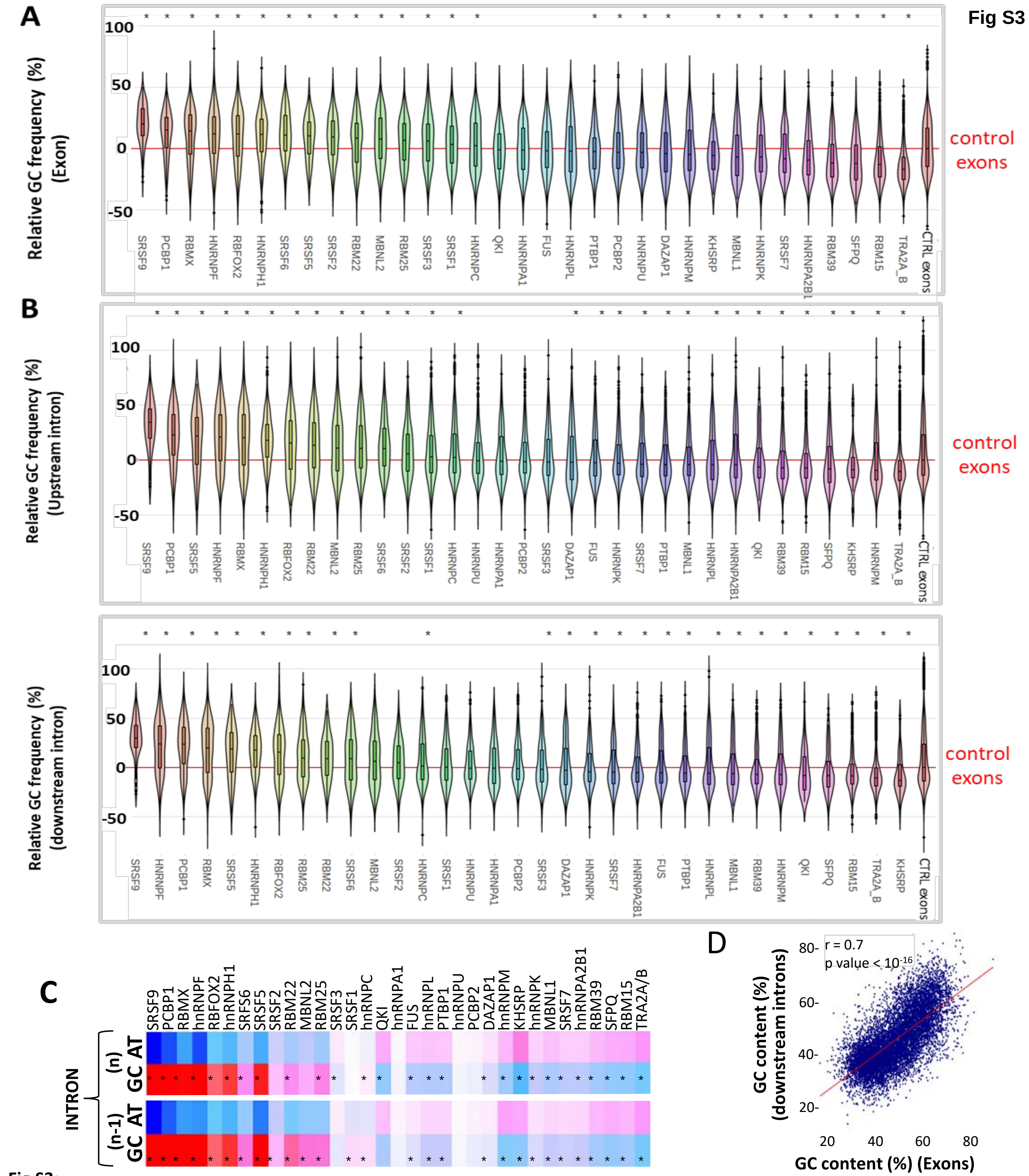

Fig S3:

a Violin plots representing the relative GC frequency in each set of splicing factor-activated exons, when compared to control exons (CTRL exons); (*) Student's test FDR < 0.05.

b Violin plots representing the relative GC frequencies in introns that are upstream and downstream, respectively, of splicing factor-activated exons (CTRL exons); (*) Wilcoxon's test FDR < 0.05.

c Heatmaps representing the relative frequency of GC and AT nucleotides in the upstream (n-1) and downstream introns of splicing factor-activated exons. (*) Wilcoxon's test, FDR < 0.05.

d Correlation between the GC content of splicing factor–activated exons and the GC content of their downstream intron; r = Pearson correlation coefficient.

Fig S4

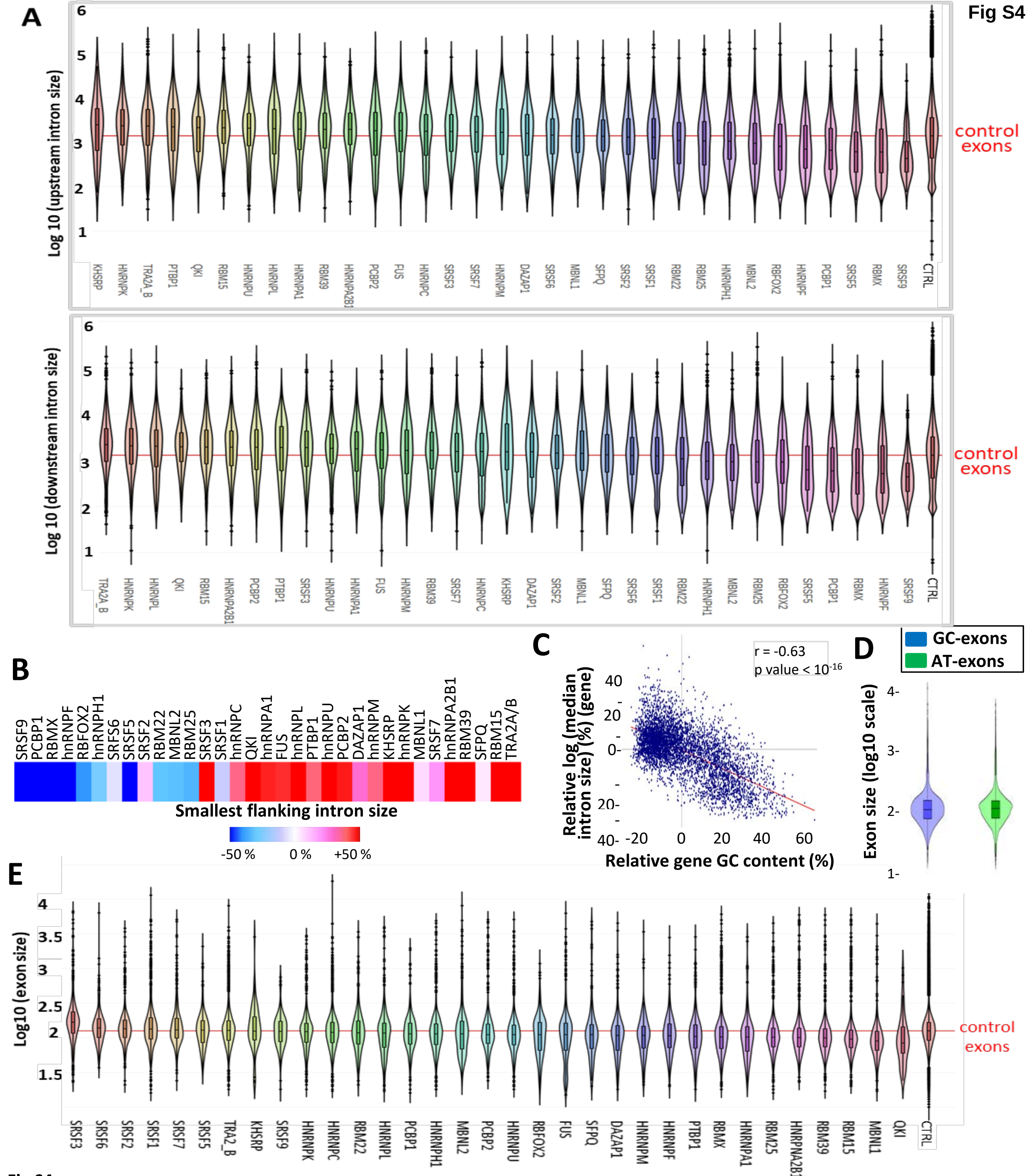

Fig S4

a Violin plots representing the relative upstream intron size (upper panel) and the relative downstream intron size (lower panel) for each set of splicing-factor activated exons, when compared to control exons (CTRL exons).

b Heatmap representing the median size of the smallest intron flanking splicing factor–activated exons, when compared to the median size of human introns. The sets of splicing factor–activated exons are represented in the same order as in fig 1a.

c Correlation between the relative median size of introns of genes hosting splicing factor-activated exons (compared to all human introns) and the relative gene GC-content (compared to all human genes); r = Pearson correlation coefficient.

d Violin plots representing the size of GC- and AT-exons.

e Violin plots representing the size of each set of splicing-factor activated exons.

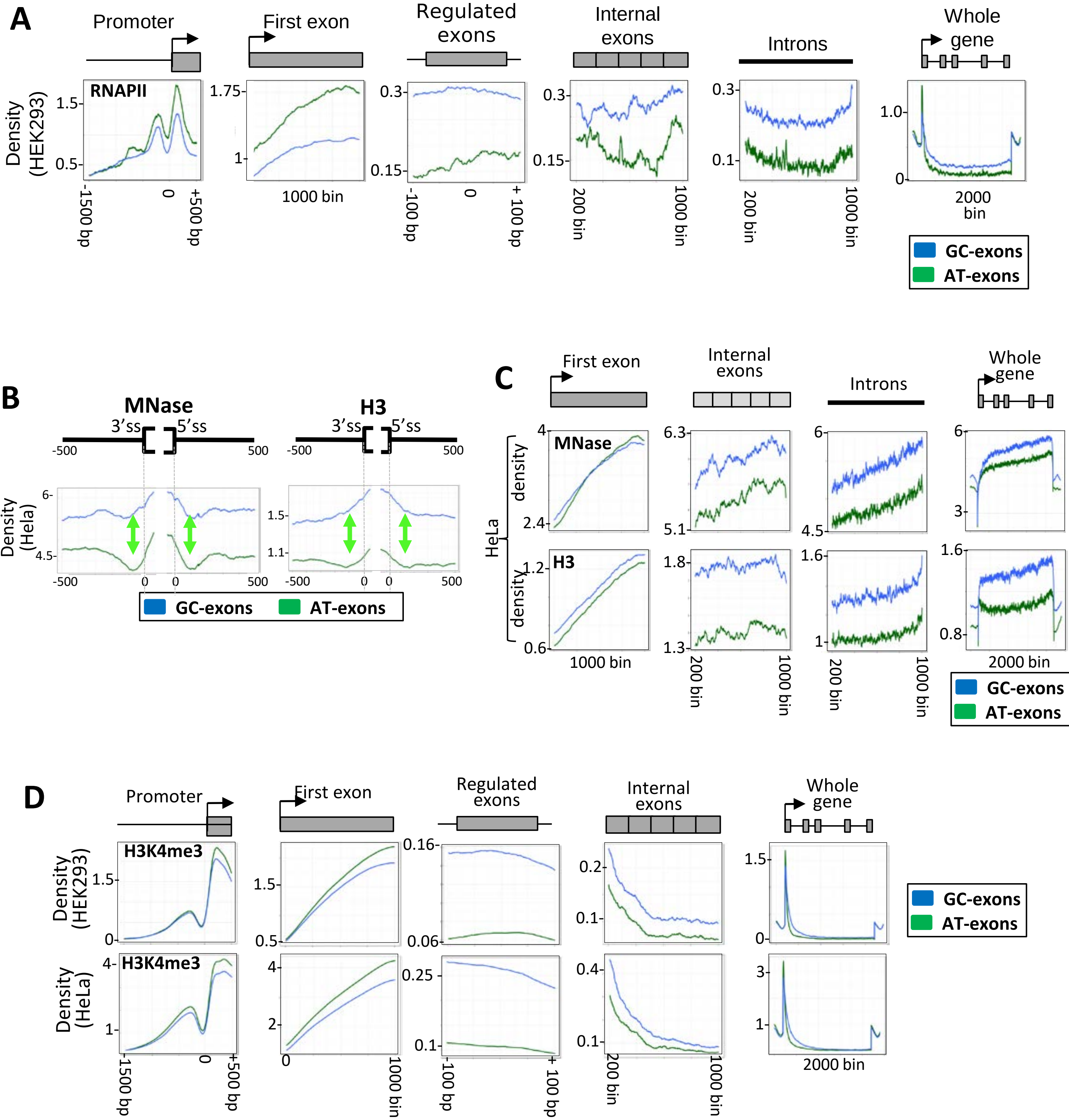

Fig S5

a Density of reads obtained after immunoprecipitation of RNAPII in HEK293 cell line and mapping to different parts of the genes hosting GC-exons or AT-exons.

b Density of reads obtained after DNA treatment with MNase (left panel) or after immunoprecipitation of the histone H3 (right panel) in Hela cells and mapping to GC-exons or AT-exons and their flanking introns.

c Density of reads obtained after DNA treatment with MNase or after immunoprecipitation of the histone H3 in Hela cells and mapping to different parts of the genes hosting GC-exon or AT-exons.

d Density of reads obtained from the HEK293 and Hela cell lines after immunoprecipitation of DNA using antibodies against H3K4me3 and H3K9ac and mapping different parts of genes hosting GC-exons or AT-exons.

Fig S6

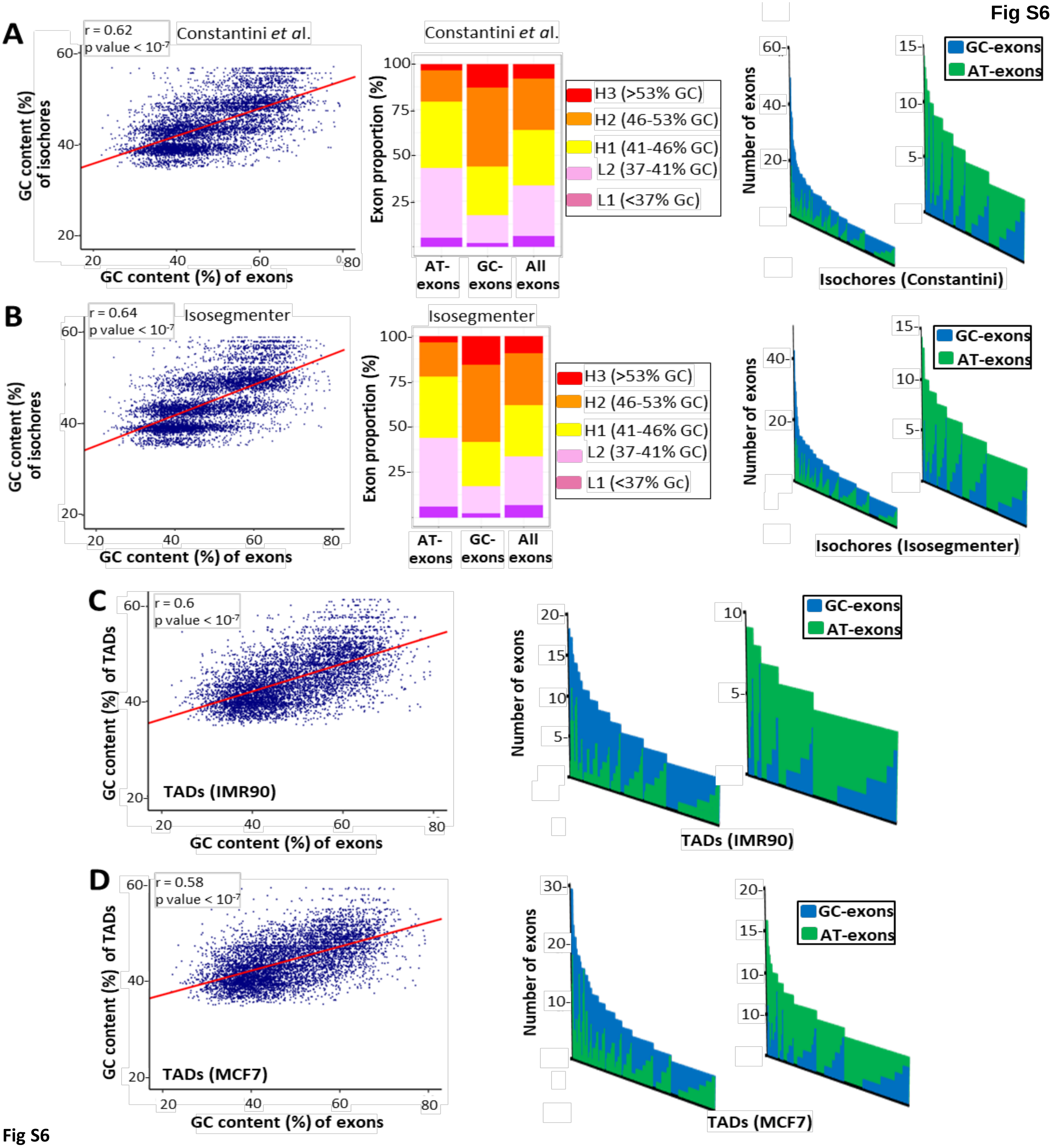

Fig S6

a Correlation between the GC content of GC-exons and AT-exons and the GC content of their hosting isochores (left panel) defined by Constantini et al (Constantini, M., Clay, O., Auletta, F. & Bernardi, G. An isochore map of human chromosomes. 2006; Genome Res 16, 536-541). Proportion of AT-exons, GC-exons, and all human exons distributed across different isochore families defined by Constantini et al. (middle panel). Number of AT-exons and GC-exons present in individual isochores defined by Constantini et al (right panel). The left and right panels, represent, isochores containing preferentially GC-exons or AT-exons, respectively.

b Same as in Supplementary Fig 6a but using isochores defined by Isosegmenter (https://github.com/bunop/isoSegmenter; Cozzi, P., Milanesi, L. & Bernardi, G. Segmenting the Human Genome into Isochores. 2015; Evolutionary bioinformatics online 11, 253-261).

c Correlation between the GC content of GC-exons and AT-exons and the GC-content of their hosting TADs defined in the IMR90 cell line (left panel). Number of AT- and GC-exons present in individual TADs annotated from the IMR90 cell line (right panel).

d Same as in Supplementary Fig 6c but using TADs defined in the MCF7 cell line.

A

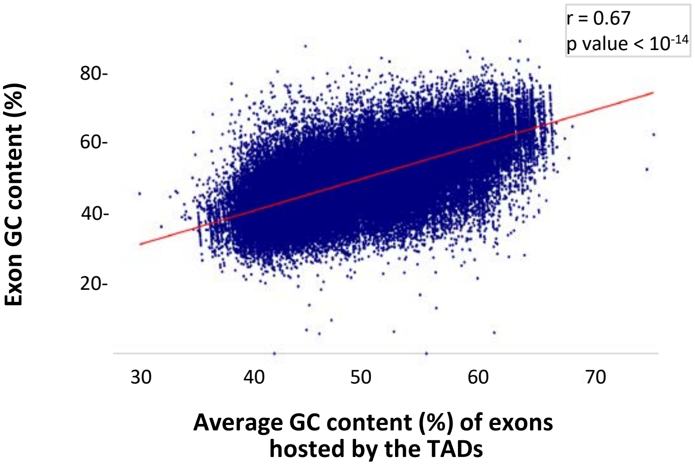

B

Model	R Formula	Function (family)	LRT test p-value (Chisq)	
			TAD K562	TAD MCF7
Full	MFE_5ss~ size_tad + (1|Tad)	lmer	$< 10^{-16}$	$< 10^{-16}$
Null	MFE_5ss ~ size_tad	lm		
Full	U1_regulated ~ size_tad + (1|Tad)	glmer (binomial)	$< 10^{-16}$	$< 10^{-16}$
Null	U1_regulated ~ size_tad	glm (binomial)		
Full	TNA_count ~ size_tad + (1|Tad)	glmer.nb	$< 10^{-16}$	$< 10^{-16}$
Null	TNA_count ~ size_tad	glm.nb		
Full	T_lc ~ size_tad + (1|Tad)	glmer.nb	$< 10^{-16}$	$< 10^{-16}$
Null	T_lc~ size_tad	glm.nb		
Full	U2_regulated ~ size_tad + (1|Tad)	glmer (binomial)	$< 10^{-16}$	$< 10^{-16}$
Null	U2_regulated ~ size_tad	glm (binomial)		

Fig S7

a Correlation between the GC content of each human exon and the average GC content of the exons hosted by the same TAD; r = Pearson correlation coefficient.

b Statistical models to test whether different splicing-related features are equally distributed between TADs.

• MFE_5’ss is a floating number vector defining, for an exon, the minimum free energy (after Anscombe transformation) of the 50-nucleotides centered on the 5’ss.

• U1_regulated is a binary vector defining if an exon is regulated by U1 (1) or not (0).

• U2_regulated is a binary vector defining if an exon is regulated by U2 (1) or not (0).

• TNA_count is an integer vector defining the number of UNA motifs found in the 50 nucleotides upstream an exon.

• T_lc is an integer vector defining the number of T-rich low complexity sequences found between the 75th and the 35th nucleotides upstream an exon.

• size_tad is an integer vector corresponding to the number of exons in each TAD.

The “Function (family)” column corresponds to the function used in R to build the model and, in parenthesis (if defined), the value for the parameter ‘family’ used in these functions. The “LRT-test” column defines the p-value of the likelihood ratio test between the null and the full models.

Fig S8

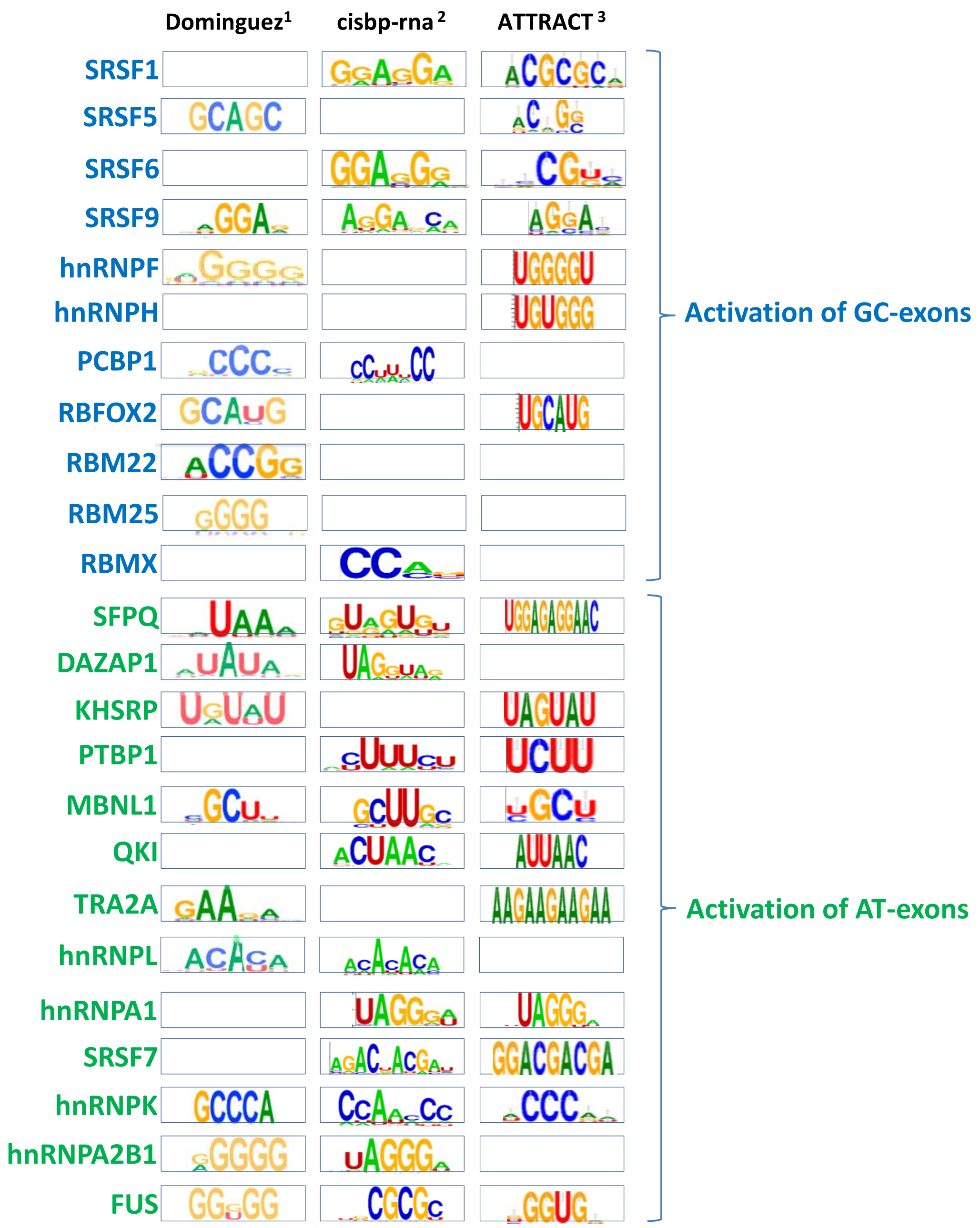

[1] Dominguez et al. PMID 29883606
[2] http://attract.cnic.es
[3] http://cisbp-rna.ccbr.utoronto.ca/index.php

Fig S8 : Splicing factor binding motifs retrieved from different resources. Splicing factors in blue color activate GC-exons, while splicing factors in green color activate AT-exons.

Fig S9

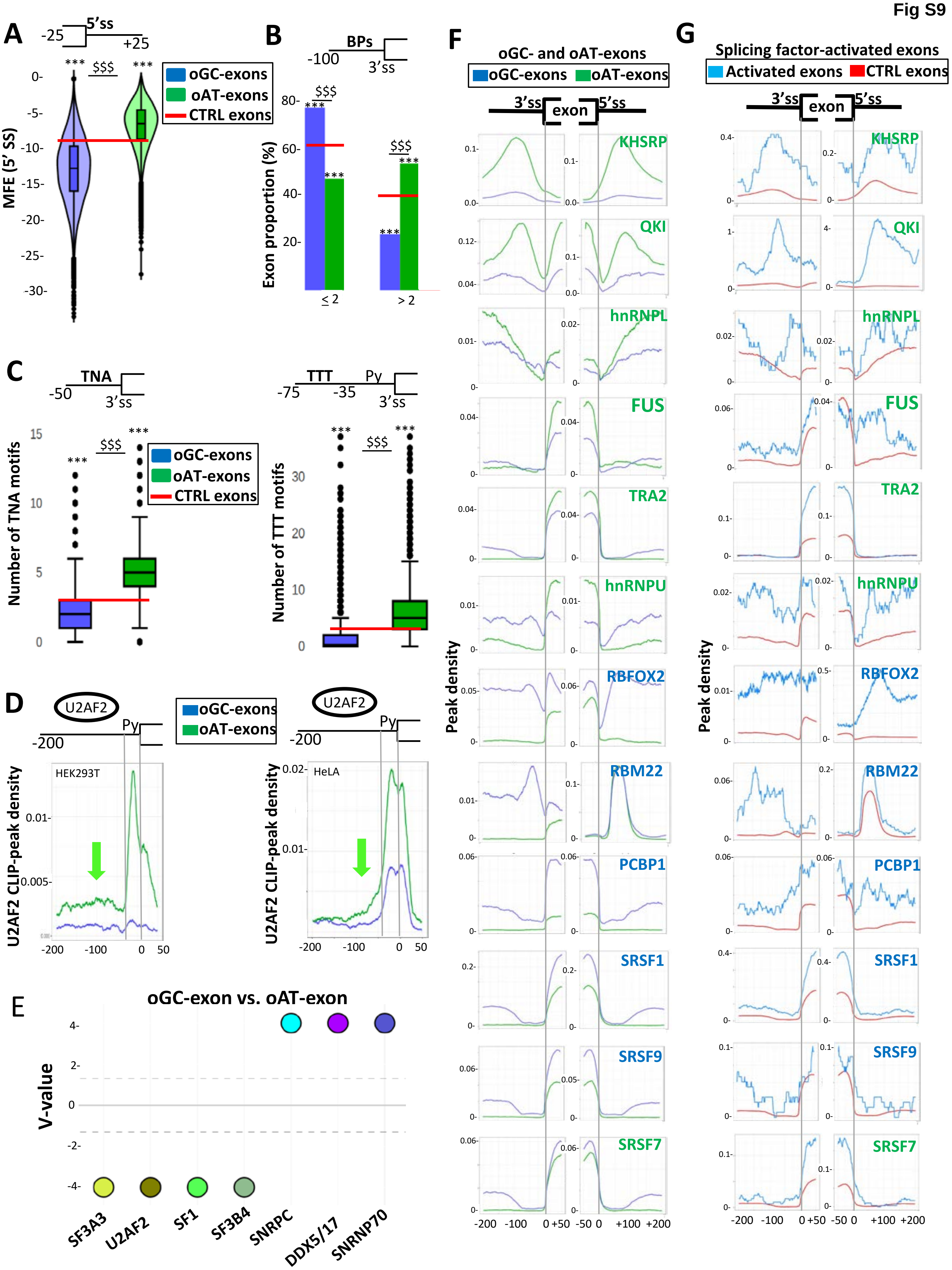
A
5'ss
-25
+25
MFE (5' SS)
oGC-exons
oAT-exons
CTRL exons
B
BPs
-100
3'ss
Exon proportion (%)
≤ 2
> 2
C
TNA
-50
3'ss
Number of TNA motifs
TTT
Py
-75
-35
Number of TTT motifs
D
U2AF2
Py
-200
HEK293T
HeLA
U2AF2 CLIP-peak density
E
oGC-exon vs. oAT-exon
V-value
SF3A3
U2AF2
SF1
SF3B4
SNRPC
DDX5/17
SNRNP70
F
oGC- and oAT-exons
3'ss
exon
5'ss
Peak density
KHSRP
QKI
hnRNPL
FUS
TRA2
hnRNPU
RBFOX2
RBM22
PCBP1
SRSF1
SRSF9
SRSF7
G
Splicing factor-activated exons
Activated exons
CTRL exons

Fig S9

a Minimum free energy (MFE) at the 5� ss of oGC-exons or oAT-exons. oGC- and oAT-exons correspond to exons that share the same characteristics (GC composition and flanking intron size) than GC- or AT-exons, respectively, but are not regulated by the set of analyzed auxiliary splicing factors. MFE was computed using 25 nucleotides within exons and 25 nucleotides within introns. The red line indicates the median value of control exons (CTRL exons). ($$$) and (***) correspond to Tukey's test FDR < 10-16 when comparing oGC-exons with oAT-exons, or when comparing oGC-exons or oAT-exons with CTRL exons, respectively.

b Proportion (%) of oGC-exons or oAT-exons with at least two or more predicted BPs in a window of 100 nucleotides in their upstream intron. The red line indicates the median value calculated for control exons (CTRL exons). (***) and ($$$) correspond to Chi2 test P < 10-16 when comparing oGC-exons with oAT-exons, or when comparing GC- exons or AT-exons with control exons, respectively.

c Boxplot representing the number of TNA sequences within the last 50 nucleotides of the upstream introns of oGC-exons and oAT-exons (left panel). Boxplot representing the number of T-rich low complexity sequences in a window between positions -35 and -75 upstream the 3'ss of oGC-exons and oAT-exons (right panel). The red lines indicate the median values of control exons (CTRL exons). ($$$) and (***) correspond to Tukey's FDR < 10-16 when comparing oGC-exons with oAT-exons, and when comparing oGC-exons or oAT-exons with control exons, respectively.

d Density of peaks obtained from publicly available U2AF2-ClIP datasets generated from HEK293T (left panel) or HeLa (right panel) cells and mapped upstream of oGC-exons and oAT-exons. The green arrows indicate reads that mapped upstream of the Py tract.

e The V-value is a representation of a P value calculated by comparing the proportion of oGC-exons and oAT-exons activated by individual spliceosome-associated factors. A V-value above the dotted line that corresponds to log10 (0.05) is statistically significant.

f Density of peaks obtained from publicly available ClIP datasets and related to different splicing factors (as indicated) in regions overlapping oGC-exons (blue curves) or oAT-exons (green curves).

g Density of peaks obtained from publicly available ClIP datasets and related to different splicing factors (as indicated) in regions overlapping control exons (CTRL, red curves) or exons activated by the indicated splicing factor (blue curves).

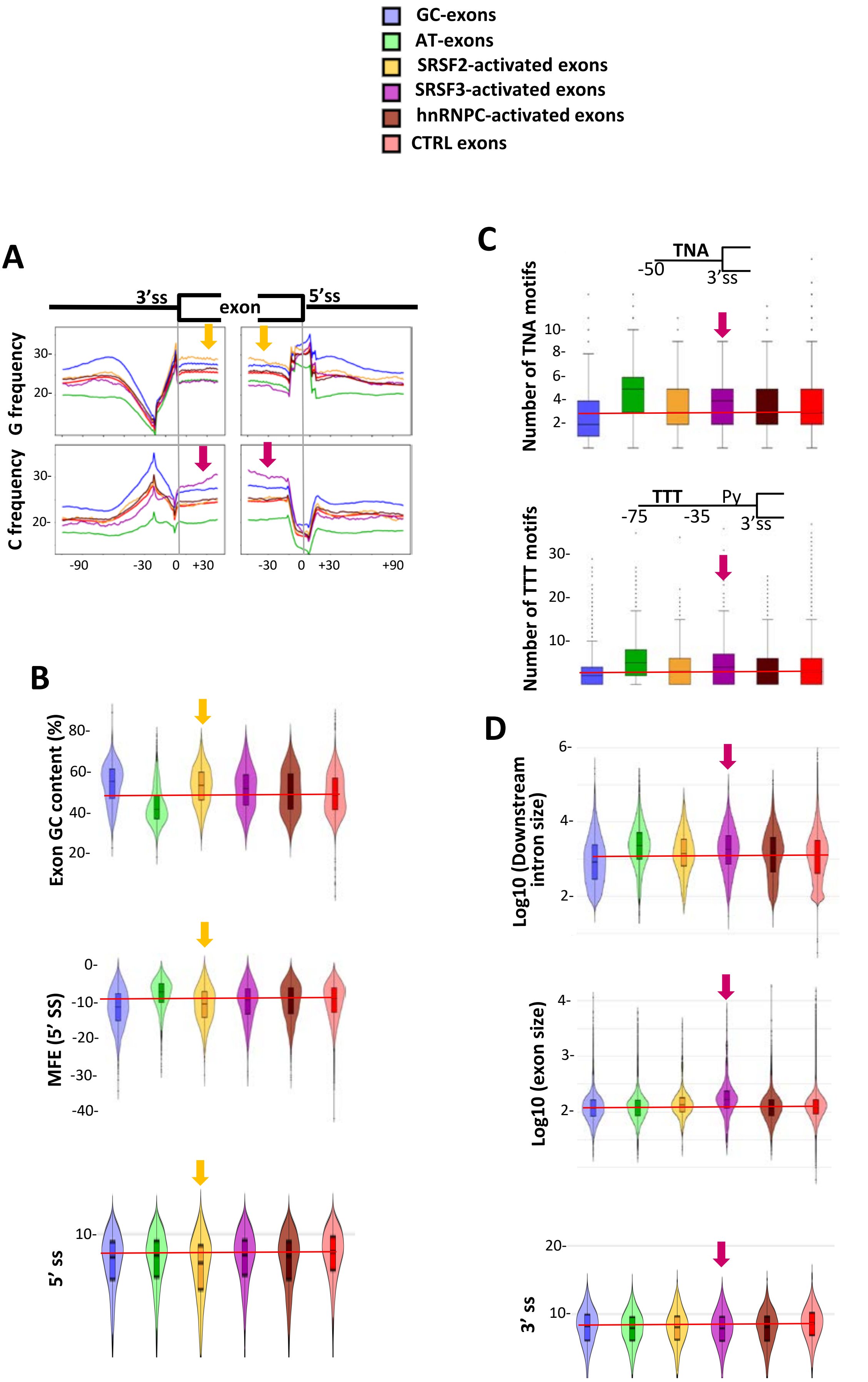
GC-exons
AT-exons
SRSF2-activated exons
SRSF3-activated exons
hnRNPC-activated exons
CTRL exons
A
3'ss
exon
5'ss
G frequency
C frequency
-90
-30
0
+30
+90
B
Exon GC content (%)
MFE (5' SS)
5' ss
C
TNA
-50
3'ss
Number of TNA motifs
TTT
Py
-75
-35
Number of TTT motifs
D
Log10 (Downstream intron size)
Log10 (exon size)
3' ss

Fig S10

a Nucleotide frequency (%) maps in different sets of exons and their flanking intronic sequences.

b Violin plots representing the GC content (%, upper panel) of GC-, AT-, and CTRL-exons, and SRSF2-, SRSF3-, and hnRNPC-activated exons. Violin plots representing the Minimum Free Energy (MFE) computed using 25 nucleotides within exons and 25 nucleotides within introns at the 5’ ss (middle panel) of GC-, AT-, and CTRL-exons, and SRSF2-, SRSF3-, and hnRNPC-activated exons.. Violin plots representing the 5’ ss score (lower panel) of GC-, AT-, and CTRL-exons, and SRSF2-, SRSF3-, and hnRNPC-activated exons. SRSF2-activated exons (orange arrows) have higher GC content, lower MFE at 5’ ss and weaker 5’ ss, when compared with SRSF3- or hnRNPC-activated exons.

c Boxplot representing the number of TNA sequences within the last 50 nucleotides of the upstream introns of GC-, AT-, and CTRL-exons, and SRSF2-, SRSF3-, and hnRNPC-activated exons. (upper panel). Boxplot representing the number of T-rich low complexity sequences in a window between positions -35 and -75 upstream the 3’ss of GC-, AT-, and CTRL-exons and SRSF2-, SRSF3-, and hnRNPC-activated exons (lower panel).

d Violin plots representing the size of introns downstream of GC-, AT-, and CTRL-exons and SRSF2-, SRSF3-, and hnRNPC-activated exons (lower panel). Violin plots representing the size of GC-, AT-, and CTRL-exons, and SRSF2-, SRSF3-, hnRNPC-activated exons. (middle panel). Violin plots representing the 3’ ss score (lower panel) of GC-, AT-, and CTRL-exons and SRSF2-, SRSF3-, and hnRNPC-activated exons. SRSF3-activated exons (purple arrows) have higher number of 3’-related splicing signal decoys, larger intronic size, and weaker 5’ ss when compared with SRSF2- and hnRNPC-activated exons.

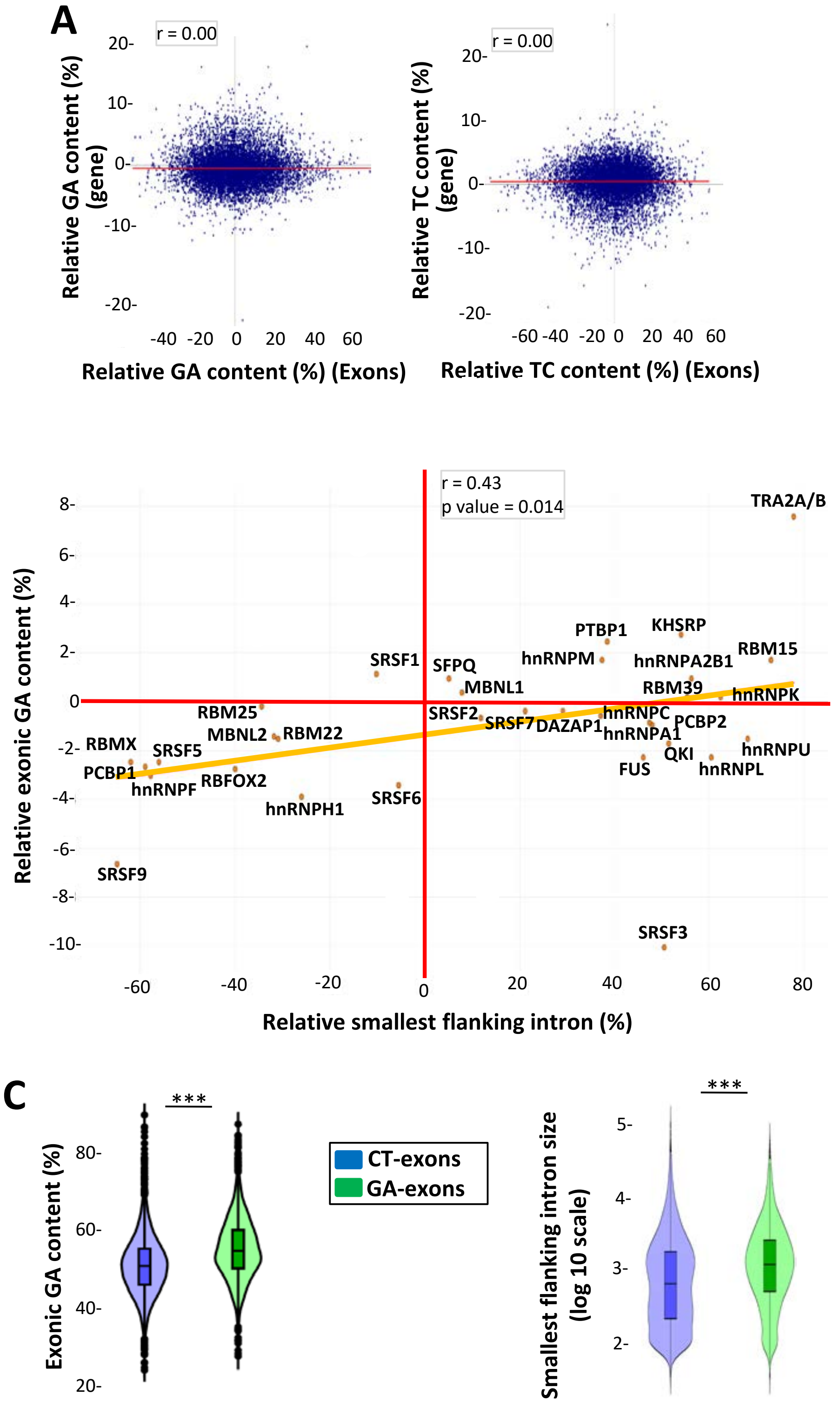

Fig S11

a Correlation between the GA- (left panel) or TC- (right panel) content of splicing factor–activated exons and the GA- or TC-content, respectively, of their hosting gene; r = Pearson correlation coefficient.

b The x-axis represents the relative median size of the smallest intron flanking splicing factor–activated exons, as compared with the median size of human introns. The y-axis represents the relative median GA content of splicing factor–activated exons, as compared with the median GA-frequency of control exons; r = Pearson correlation coefficient.

c Violin plots representing the GA content (%) of GA-exons and CT-exons (left panel) and the size of the smallest intron flanking GA-exons and CT-exons (right panel). (***) correspond to Wilcoxon's test P < 10-16 when comparing GC-exons with AT-exons.

Fig S12

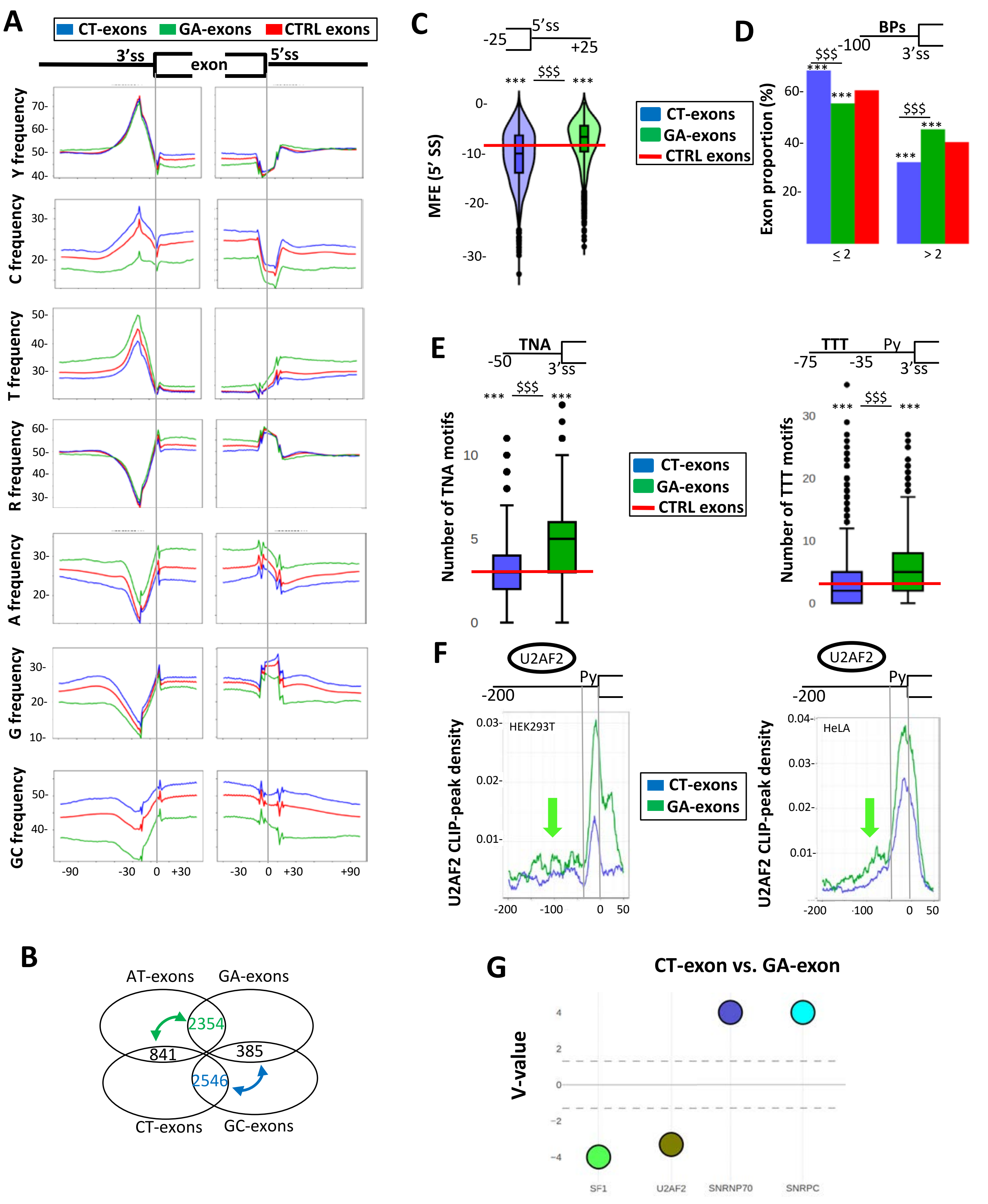
A
CT-exons
GA-exons
CTRL exons
3'ss
exon
5'ss
Y frequency
C frequency
T frequency
R frequency
A frequency
G frequency
GC frequency
-90
-30
0
+30
+90
B
AT-exons
GA-exons
2354
841
385
2546
CT-exons
GC-exons
C
5'ss
-25
+25
$$$
***
MFE (5' SS)
D
BPs
-100
3'ss
Exon proportion (%)
≤ 2
> 2
E
TNA
-50
3'ss
Number of TNA motifs
TTT
Py
-75
-35
Number of TTT motifs
F
U2AF2
Py
-200
U2AF2 CLIP-peak density
HEK293T
HeLA
-100
50
G
CT-exon vs. GA-exon
V-value
SF1
U2AF2
SNRNP70
SNRPC

Fig S12

a Nucleotide frequency (%) maps in different sets of exons and their flanking intronic sequences.

b Number of AT-exons belonging to the sets of GA- or CT-exons and number of GC-exons belonging to the sets of GA- or CT-exons.

c Minimum free energy (MFE) at the 5'ss of GA-exons or CT-exons. The red line indicates the median value of control exons (CTRL exons). ($$$) and (***) correspond to Tukey's test FDR < 10-16 when comparing GA-exons with CT-exons, or when comparing GA-exons or CT-exons with control exons, respectively.

d Proportion (%) of GA-exons, CT-exons or control exons with at least two or more that two predicted BPs in a window of 100 nucleotides in their upstream intron. (***) and ($$$) correspond to Chi2 test P < 10-7 when comparing GA-exons with CT-exons, or when comparing GA- exons or CT-exons with control exons, respectively.

e Boxplot representing the number of TNA sequences within the last 50 nucleotides of the upstream introns of GA-exons and CT-exons (left panel). Boxplot representing the number of T-rich low complexity sequences in a window between positions -35 and -75 upstream the 3'ss of GA-exons and CT-exons (right panel). The red lines indicate the median values of control exons. ($$$) and (***) correspond to Tukey's FDR < 10-16 when comparing GA-exons with CT-exons and when comparing GA-exons or CT-exons with control exons, respectively.

f Density of peaks obtained from publicly available U2AF2-ClIP datasets generated from HEK293T (left panel) or HeLa (right panel) cells and mapped upstream of GA-exons and CT-exons. The green arrows indicate reads that mapped upstream of the Py tract.

g The V-value is a representation of a P value calculated by comparing the proportion of CT-exons and GA-exons activated by SF1, U2AF2, SNRP70, or SNRPC. A V-value above the dotted line that corresponds to log10 (0.05) is statistically significant.
