# Supplementary material for: Characterizing the interplay between gene nucleotide composition bias and splicing
Source: Genome Biol. 2019 Nov 29;20:259. doi: 10.1186/s13059-019-1869-y (PMC6883713; doi:10.1186/s13059-019-1869-y)
Supplement: Supplementary file 5 — Additional file 5. Review history. [file 13059_2019_1869_MOESM5_ESM.docx]

Review History

**First round of review**

**Reviewer 1**

**Are you able to assess all statistics in the manuscript, including the appropriateness of statistical tests used?**

Yes, and I have assessed the statistics in my report.

**Comments to author:**

Lemaire, Fontrodona et al present a fundamental link between the splicing process and the nucleotide composition of exons, their surrounding introns and the gene overall. Using nucleotide composition the authors define AT-exons and GC-exons and reveal a number of fundamental properties that distinguish such classes, including the control by a variety of splicing factors. The authors furthermore link sequence composition and splicing status to chromatin variables (nucleosome occupancy, histone tail modifications and polymerase occupancy) as well as large-scale chromatin organization (e.g. topologically-associated domains) and intron length.

Overall, the work is rather correlative in nature. However, the uncovered correlations are indeed very interesting and could spur a lot of new thought in the field. Therefore, I believe the manuscript is generally well-suited for Genome Biology.

I have a number of concerns that the authors should take into account. Overall, I do think that it is feasible to remedy these concerns.

A. Major Concerns:

1. The observation of differences in gene structure between high and low-GC isochores is partially known (although not clearly articulated in a mechanistic context): Gene prediction programs, such as GENSCAN, Fgenesh and Geneid (among others) employ different parameter sets in high-GC isochores and low-GC isochores to decide what is an exon and what is not. This was done based on the realization that sequences characteristic of exons and introns are not identical in different isochores. This work needs to be cited and the authors should frame their research as revealing the splicing-regulatory characteristics that underlie the above observations.

2. In the definition of GC and AT-rich exons (page 5, top paragraph) the authors do not seem to discuss exon size. Two sides of the same coin include the possibility that 1) microexons (see Irimia et al, Cell 2014) are enriched in one of the two classes and 2) that shorter exons have more variable GC content, simply because the lower number of nucleotides does not allow an exact definition. Potentially, considering exon size could change some of the observations.

3. The manuscript is based in large parts based on two classes of exons: GC rich and AT rich ones. This, strand-independent measure, already leads intuitively to chromatin-related variables, which is then nicely shown in the manuscript. However, there are other (strand-dependent) ways to classify exons, for example according to GA-content, which correlates with specific splicing factors being able to recognize splicing enhancers. It is possible that some of the characteristics that differentiate GC and AT rich exons, would even better separate GA and CT rich exons. I'd like to see if GA and CT rich exons have an even stronger differential behavior in terms of predicted secondary structures at 5'ss and dependency on U1/U2-snRNP-associated proteins. Whatever the outcome, the authors should mention this clearly.

4. Discussion (Page 10, first paragraph). Previous research (Tilgner et al, Genome Research) has linked exonic GC to co-transcriptional splicing (and shown that splicing happens predominantly during transcription). This should be mentioned and discussed here.

5. page 9, end of results. The authors mention a correlation of exonic GC content and TAD GC content. Given the other findings of the manuscript, one could speculate that exons in the same TAD follow similar rules. The authors could develop this thought a bit deeper, if their data supports it.

B. Minor Concerns

1. page 3, line 8: The authors mention that secondary structures can influence splicing. An extra phrase explaining how much is known about the frequency of this phenomenon.

2. page 4, line 24. The authors seem to refer to published RNAseq datasets (following splicing factor manipulations). Judging from the accession numbers, there is lots of ENCODE data, but also that of smaller labs. There should be citations in the text, especially for smaller labs.

3. page 4, line 28. Splicing factor activated exons presumably include indirect activation effects. This caveat should be well explained.

4. page 5, line 32: Free energy calculations appear to have been calculated using the Vienna RNA package. This needs to be cited appropriately in the main text.

5. page 6, line 7. Branch point prediction appear to have been carried out using software by Corvelo et al. This needs to be cited in the main text.

6. page 6, line 27. Splicing factor-KD papers need to be cited in the main text.

**Reviewer 2**

**Are you able to assess all statistics in the manuscript, including the appropriateness of statistical tests used?**

Yes, and I have assessed the statistics in my report.

**Comments to author:**

This manuscript by Lemaire et al investigates how exonic GC content and the surrounding genomic environment contributes to splicing factor-dependent exon recognition. They initially selected a set of exons whose inclusion requires the expression of at least one splicing factor (selected from RNAseq datasets in which splicing factors have been overexpressed or knocked down). Certain splicing factors played a more significant regulatory role in exons that were GC-rich, while others showed a preference for regulating AT-rich exons. The authors define two groups for further analysis: (1) "GC-exons", which are GC-rich with short flanking introns and (2) "AT-exons," which are AT-rich with long flaking introns (relative to "control exons"). The GC composition of flanking introns, genes, and isochores surrounding these two groups mirrored the GC composition of the exons in each group. Exonic nucleotide bias was linked to more predicted secondary structure at the 5'SS and a dependence on U1-associated splicing factors for GC-rich exons. Increased numbers of branch points at the 3'SS and an increased dependence of U2-associated splicing factors were associated with AT-rich exons.

The latter two observations are interesting, although they arguably provide limited insights about / formal proof of the underlying mechanisms. Other observations are rather descriptive and/or not unexpected given known nucleotide composition biases and previous literature. For these reasons, in our opinion the manuscript would greatly benefit from additional analyses to justify publication in Genome Biology.

Main points:

1. Given the known nature and size of isochores, it is not surprising that local genomic nucleotide composition bias affects similarly exons and introns, longer genomic regions and even their 3D genomic organization. Along with previous associations -properly referenced by the authors, sentences /conclusions as the following are hardly surprising: "splicing factor-activated GC- and AT-rich exons were flanked by GC- and AT-rich intronic sequences"," The GC-exon group depends on splicing factors activating GC-rich exons that are flanked by small introns, while the AT-exon group depends on splicing factors activating AT-rich exons that are flanked by large introns", "We found that exons and their flanking intronic sequences had similar nucleotide composition biases when considering both whole intronic sequences (Fig. 1b) or intronic sequences located just upstream or downstream exons","GC-exons were impoverished in Ts but enriched in Cs just upstream 1 of their 3' ss as compared to control exons", " there was a positive correlation between the GC content of exons and the GC content of their hosting gene", "exons embedded in a GC-rich environment have a higher nucleosome density.", "there was a marked nucleosome-free region both upstream and downstream of AT-exons when compared to GC-exons", " there was a positive correlation between the GC content of exons and the GC content of their hosting isochores", " a positive correlation between the GC content of exons and the GC content of the TAD they belong to".

2. A number of methodological issues need to be addressed more thoroughly, including:

- The definitions of the groups of GC-exons and AT-exons should be more rigorous, including the threshold values used for considering a flanking intron to be "short" or "long".

- Similarly, the set of exons referred to as "control exons" should be more clearly defined. For example, on p. 13 of the methods section it is stated that "the control set of exons (typo: s is missing in the text) used corresponds to human exons annotated in FASTERDB." Is this the entire set of annotated human exons or a subset of exons randomly selected to match the number of exons contained in GC- and AT-exon sets that were being compared?

- What is the overlap between the "U1-exons" and the "GC-exons" or the "AT-exons?" Likewise, how much overlap is there between the "U2-exons" and the GC- or AT-exon sets?

- Why were the datasets involving "spliceosome associated factors" from Figure 3 not considered as a part of Figure 1 to define the "GC-exon" and "AT-exon" sets?

- More generally, the authors initially used published datasets in which splicing factors had been knocked-down or overexpressed to choose the groups of "GC-exons" and "AT-exons." However, when they went on to look at the surrounding GC/AT environment in genes/isochores/TADS, why did they limit this analysis to this same set of ~7000 exons. Likely, many more exons are alternatively spliced, regulated by splicing factors, and fit the same criteria used to select the GC and AT exons even though they don't happen to be a part of the RNAseq datasets involving knockdown of select splicing factors. Could the authors look at an expanded set of GC-rich and AT-rich exons to analyze the GC content of their genomic environment? That way they could show that their assertion is not limited to these ~7000 exons but is a more general characteristic of GC-rich or AT-rich exons. They could then look at iCLIP data to evaluate if splicing factors are binding the expanded set of exons as predicted.

3. Key insights of the manuscript rely on predictions (RNA secondary structures for 5' ss in GC-rich exons, branchpoints associated with AT-rich exons) that have limited value in the absence of functional data, but that the authors raise to the level of conclusions present even in the abstract. It would greatly enhance the value of the manuscript if the authors would make an effort to experimentally test some of their predictions/conclusions. For example, could they assess the existence of secondary structures in one or two examples of GC-rich exons using minigenes, proving for example that disruption / compensatory mutations of predicted base pairing affects exon inclusion and that dependence on DDX5/17 is no longer there when the secondary structures are disrupted? Similar experiments could be designed to address the influence of the existence of potential branch sites in the inclusion of AT-rich exons. More generally, the authors could make use of recent branch point mapping efforts (e.g. Mercer et al 2015) to substantiate the real use of predicted branchpoints.

4. The connections between epigenetic marks / 3D organization and exon recognition are particularly weak. For example, are the histone marks that are more prevalent for the "GC-exons" a consequence of open chromatin related to the higher levels of transcription associated with these exons, or is it related to splicing regulation? The conclusions drawn from this panel are unclear— "Therefore, GC-exons and AT-exons…are embedded in different chromatin environments." What is the significance of this?

Additional points:

a) In Figure 1c, exons dependent on SRSF2, SRSF3, and hnRNPC were enriched in GC content, but they were excluded from the "GC-exon" set because their flanking introns were not shorter than controls. It would be interesting to compare this set to the GC-rich + short intron group, since this could help to discriminate between the role of GC-content and flanking intron size, as well as possibly provide potential insights as to why SRSF2, SRSF3 and hnRNPC-dependent exons display different features.

b) Several datasets examining SF3B1 knockdown are mentioned as being analyzed in the supplemental material, but the results of these analyses are not mentioned. Could the authors please explain this?

c) Page 7, line 31. It is unclear what "In this setting…" in the beginning of the paragraph is referring to.

d) Page 6: the sentence "the Py tract of GC-exons was similar to that of control exons" may be misleading, as Us provide higher binding affinity to U2AF2 than Cs and, for the same reason, the conclusion that "the analysis of U2AF2 CLIP-Seq datasets revealed a higher U2AF2-related signal upstream of AT-exons, as compared to GC-exons (Fig. 2h)" is not unexpected.

**Authors Response**

**Point-by-point responses to the reviewers’ comments:**

“Reviewer #1: Lemaire, Fontrodona et al present a fundamental link between the splicing process and the nucleotide composition of exons, their surrounding introns and the gene overall. Using nucleotide composition the authors define AT-exons and GC-exons and reveal a number of fundamental properties that distinguish such classes, including the control by a variety of splicing factors. The authors furthermore link sequence composition and splicing status to chromatin variables (nucleosome occupancy, histone tail modifications and polymerase occupancy) as well as large-scale chromatin organization (e.g. topologically-associated domains) and intron length.

Overall, the work is rather correlative in nature. However, the uncovered correlations are indeed very interesting and could spur a lot of new thought in the field. Therefore, I believe the manuscript is generally well-suited for Genome Biology. I have a number of concerns that the authors should take into account. Overall, I do think that it is feasible to remedy these concerns. “

*Response: We thank the Reviewer for her/his very positive and constructive comments. While some of the results of our manuscript may appear as “correlative in nature”, we would like to emphasize that the predictions based on the analyses of nucleotide composition biases are experimentally supported by different kinds of large-scale datasets. As an example, the analysis of the sequences of exons whose regulation by splicing factors was revealed by experimental datasets (Fig. 1 and 2), led us to hypothesize that exons embedded in GC-rich context depend on U1-related factors, while exons in an AT-rich context depend on U2-related factors. We showed using RNA-seq and CLIP-seq datasets that this is indeed the case (Fig. 3). Similarly, the hypothesis that exons sharing the same nucleotide composition biases are not randomly distributed in the human genome was supported by experimental datasets (ChIP-seq and TAD annotations, Fig. 4, 5, and 6). Finally, we performed a couple of experiments requested by Reviewer #2 to provide some mechanistic insights (see new Fig. 4 and Reviewer #2, Main point #3).*

“A. Major Concerns:

1. The observation of differences in gene structure between high and low-GC isochores is partially known (although not clearly articulated in a mechanistic context): Gene prediction programs, such as GENSCAN, Fgenesh and Geneid (among others) employ different parameter sets in high-GC isochores and low-GC isochores to decide what is an exon and what is not. This was done based on the realization that sequences characteristic of exons and introns are not identical in different isochores. This work needs to be cited and the authors should frame their research as revealing the splicing-regulatory characteristics that underlie the above observations.”

*Response: We thank the Reviewer for this comment, which is important since it has already been observed that gene GC-content variation is associated with specific gene features, having consequences on computational prediction of exon and splicing sites, as now better highlighted in the Introduction (page 4, line 2).*

“2. In the definition of GC and AT-rich exons (page 5, top paragraph) the authors do not seem to discuss exon size. Two sides of the same coin include the possibility that 1) microexons (see Irimia et al, Cell 2014) are enriched in one of the two classes and 2) that shorter exons have more variable GC content, simply because the lower number of nucleotides does not allow an exact definition. Potentially, considering exon size could change some of the observations.”

*Response: As requested by the Reviewer, we analyzed exon size and observed that, in general, exons depending on splicing factors were smaller than the average size of all human exons, with the exception of SRSF-depending exons and particularly SRSF3-dependent exons (new Supplementary Fig. 4e). This observation is consistent with previous reports showing that alternative exons have often a smaller size than constitutive exons. In contrast to the negative correlation that we observed between the GC content of exons and the size of their flanking introns (Fig 1c, upper panel), there was no correlation between the GC content of exons and exon size (new Fig 1c, lower panel). Therefore, GC- and AT-exons had similar size (new Supplementary Fig. 4d) while they were flanked by introns of different sizes (Fig. 1e, right panel). This suggests that gene/exon GC content is not associated with constraints on exon size as opposed to intron size (page 5, line 17).*

*We must point out that our FARLINE pipeline of RNAseq analysis has not been optimized to identify micro-exons (<27 nucleotides). Of the ~10,000 exons that FARLINE detects depending on at least one analyzed splicing factor, only 139 have a size <27 nucleotides. Therefore, to address the Reviewer's question related to micro-exons, we analyzed the GC content of micro-exons identified by Irimia et al. As anticipated by the Reviewer, micro-exons had a very heterogeneous GC composition (suggesting that the lower number of nucleotides does not allow an exact definition). But the important point is that there were about 1.6 times more AT-like micro-exons (i.e., micro-exons with a GC content lower than 49,3% and with flanking introns longer than 691 nucleotides) than GC-like micro-exons (i.e., micro-exons with a GC content higher than 49,3% and with one flanking introns shorter than 691 nucleotides), which is quite similar to what we observed for regular exons (>27 nucleotides) analyzed in our manuscript (4000 AT-exons vs 3200 GC-exons). Therefore, we cannot conclude that micro-exons belong more frequently to one or the other AT- or GC-like category.*

“3. The manuscript is based in large parts based on two classes of exons: GC rich and AT rich ones. This, strand-independent measure, already leads intuitively to chromatin-related variables, which is then nicely shown in the manuscript. However, there are other (strand-dependent) ways to classify exons, for example according to GA-content, which correlates with specific splicing factors being able to recognize splicing enhancers. It is possible that some of the characteristics that differentiate GC and AT rich exons, would even better separate GA and CT rich exons. I'd like to see if GA and CT rich exons have an even stronger differential behavior in terms of predicted secondary structures at 5'ss and dependency on U1/U2-snRNP-associated proteins. Whatever the outcome, the authors should mention this clearly.”

*Response: We thank the Reviewer for raising this question, which allows us to point out that the GC/AT composition bias has a particular significance in comparison with purine/pyrimidine (GA/CT) composition bias. Indeed, we show that:*

*i) There is no correlation between the purine (or pyrimidine) content of exons and that of their flanking introns or their hosting genes (new Fig. 1b, lower panel, and Supplementary Fig. 11a) contrasting with the positive correlation between the GC-content of exons and that of their flanking introns and hosting genes (Fig. 1b, upper panel, Fig. 5a).*

*ii) The variation in exon purine-content is relatively small when comparing sets of exons regulated by different splicing factors to the average of purine frequency in all human exons (+/-8%, new Supplementary Fig. 11b), while a larger variation in the exon GC-content was observed (+/-20%, Fig. 1d). It is interesting to note that the purine content of exons has been proposed to be constrained by the organization of the genetic code. Indeed, the most frequent amino acids in proteins and most amino acids adapted to protein-physicochemical constraints (folding, solubility...) correspond to purine-rich codons (e.g., Ponce de Leon, M., et al. (2014) “The Purine Bias of Coding Sequences is Determined by Physicochemical Constraints on Proteins”. Bioinform Biol Insights 8, 93-108). Therefore, the genetic code properties could constrain the purine composition of exons compared with introns and its variation in exons.*

*iii) We noticed that pyrimidine-rich exons are strongly enriched in Cs (and also in Gs) but are actually slightly impoverished in Ts (new Supplementary Fig. 12a). Meanwhile, purine-rich exons are strongly enriched in As (and also in Ts) but are slightly impoverished in Gs (new Supplementary Fig. 12a). As a consequence, pyrimidine-rich exons share properties with GC-rich exons, while purine-rich exons share properties with AT-rich exons (Supplementary Fig. 11c and 12).*

*Two important observations must be highlighted in order to interpret these results. First, as already mentioned, there was a correlation between the GC content of exons and that of their flanking introns (and their hosting genes). In contrast, there was no correlation between the GA content of exons and that of their flanking introns, nor that of their hosting genes. Second, while it is well established that the 1D organization of some genomes (in particular the human genome) relies on the succession of regions differentially enriched in GC nucleotides (i.e., isochores), no purine- or pyrimidine-dependent global genomic organization has been reported to the best of our knowledge. Therefore, observations made on purine/pyrimidine composition biases confirmed the conclusions of our manuscript: the GC-based genome organization is associated with gene- and exon-GC composition biases, and therefore on RNA-GC composition biases with consequences on splicing (page 5, line 13 and page 13, line 1).*

“4. Discussion (Page 10, first paragraph). Previous research (Tilgner et al, Genome Research) has linked exonic GC to co-transcriptional splicing (and shown that splicing happens predominantly during transcription). This should be mentioned and discussed here.”

*Response: We thank the Reviewer for mentioning the manuscript of Tilgner et al reporting that introns flanking GC-rich exons tend to be spliced more efficiently. This observation agrees with articles showing that GC-rich genomic regions are in contact with speckles and that exons of the same gene are spliced according to the same efficiency and mode (see Discussion). These observations and our work support a model where GC-rich genes that are hosted by GC-rich TADs are actively transcribed, have small introns, and their transcripts are spliced efficiently and probably co-transcriptionally. We added this point in the Discussion section (page 14, line 1).*

“5. page 9, end of results. The authors mention a correlation of exonic GC content and TAD GC content. Given the other findings of the manuscript, one could speculate that exons in the same TAD follow similar rules. The authors could develop this thought a bit deeper, if their data supports it.”

*Response: We added a new figure showing that the GC content of any human exons correlated with the average GC content of the exons hosted by the same TAD (Supplementary Fig. 7a). We also developed statistical models showing that: i) splicing-related values (e.g., MFE values at 5’ss, number of decoys of 3’ splicing signals) were not randomly distributed among TADs and ii) exons regulated by U1 (or U2) were more concentrated in some TADs than in others (Supplementary Fig. 7b). Collectively, these results support the notion that exons in the same TAD follow similar rules (page 10, line 7).*

B. Minor Concerns

“1. page 3, line 8: The authors mention that secondary structures can influence splicing. An extra phrase explaining how much is known about the frequency of this phenomenon.”

*Response: The description of the role of secondary structures in splicing was developed as requested (p.3, l.6).*

“2. page 4, line 24. The authors seem to refer to published RNAseq datasets (following splicing factor manipulations). Judging from the accession numbers, there is lots of ENCODE data, but also that of smaller labs. There should be citations in the text, especially for smaller labs.”

*Response: We thank the Reviewer for bringing this important aspect to our attention and the references were added in the manuscript (page 4, line 26 and page 6, line 35) and in Table Sup S1.*

“3. page 4, line 28. Splicing factor activated exons presumably include indirect activation effects. This caveat should be well explained.”

*Response: As requested, we addressed this concern in the manuscript (page 12, line 24) and showed by using publicly available ClIPs that splicing factors were enriched on or near the exons that they regulated (new Supplementary Fig. 9g).*

“4. page 5, line 32: Free energy calculations appear to have been calculated using the Vienna RNA package. This needs to be cited appropriately in the main text.”

*Response: The manuscript was modified accordingly (page 6, line 1).*

“5. page 6, line 7. Branch point prediction appear to have been carried out using software by Corvelo et al. This needs to be cited in the main text.”

*Response: The manuscript was modified accordingly (page 6, line 13).*

“6. page 6, line 27. Splicing factor-KD papers need to be cited in the main text.”

*Response: The manuscript was modified accordingly (page 6, line 35).*

“Reviewer #2: This manuscript by Lemaire et al investigates how exonic GC content and the surrounding genomic environment contributes to splicing factor-dependent exon recognition. They initially selected a set of exons whose inclusion requires the expression of at least one splicing factor (selected from RNAseq datasets in which splicing factors have been overexpressed or knocked down). Certain splicing factors played a more significant regulatory role in exons that were GC-rich, while others showed a preference for regulating AT-rich exons. The authors define two groups for further analysis: (1) "GC-exons", which are GC-rich with short flanking introns and (2) "AT-exons," which are AT-rich with long flaking introns (relative to "control exons"). The GC composition of flanking introns, genes, and isochores surrounding these two groups mirrored the GC composition of the exons in each group. Exonic nucleotide bias was linked to more predicted secondary structure at the 5'SS and a dependence on U1-associated splicing factors for GC-rich exons. Increased numbers of branch points at the 3'SS and an increased dependence of U2-associated splicing factors were associated with AT-rich exons.

The latter two observations are interesting, although they arguably provide limited insights about / formal proof of the underlying mechanisms. Other observations are rather descriptive and/or not unexpected given known nucleotide composition biases and previous literature. For these reasons, in our opinion the manuscript would greatly benefit from additional analyses to justify publication in Genome Biology.”

*Response: We thank the Reviewer for the positive and constructive comments. We understood from some of the Reviewer’s concerns that we did not sufficiently explained and highlighted some of our conclusions. In addition, addressing both Reviewer’s concerns allowed us to improve the manuscript. We also performed additional experiments as requested by the Reviewer.*

“Main points:

1. Given the known nature and size of isochores, it is not surprising that local genomic nucleotide composition bias affects similarly exons and introns, longer genomic regions and even their 3D genomic organization. Along with previous associations -properly referenced by the authors, sentences /conclusions as the following are hardly surprising: "splicing factor-activated GC- and AT-rich exons were flanked by GC- and AT-rich intronic sequences"," The GC-exon group depends on splicing factors activating GC-rich exons that are flanked by small introns, while the AT-exon group depends on splicing factors activating AT-rich exons that are flanked by large introns", "We found that exons and their flanking intronic sequences had similar nucleotide composition biases when considering both whole intronic sequences (Fig. 1b) or intronic sequences located just upstream or downstream exons","GC-exons were impoverished in Ts but enriched in Cs just upstream 1 of their 3' ss as compared to control exons", " there was a positive correlation between the GC content of exons and the GC content of their hosting gene", "exons embedded in a GC-rich environment have a higher nucleosome density.", "there was a marked nucleosome-free region both upstream and downstream of AT-exons when compared to GC-exons", " there was a positive correlation between the GC content of exons and the GC content of their hosting isochores", " a positive correlation between the GC content of exons and the GC content of the TAD they belong to".”

*Response: We understand the Reviewer's feeling who is somehow considering that we “are pushing open doors”. Nevertheless, we provided Reviewer #1 with new elements showing that the points we addressed in the manuscript were not "obvious" and were important to address. For example, if there was a correlation between the GC content of exons and that of their flanking introns, there was no correlation between the purine composition of exons and that of their flanking introns (e.g., new Fig. 1b, upper vs. lower panel). Similarly, a correlation between GC-content and intron size was observed, but this correlation did not exist when considering exon size (e.g., new Fig. 1c, upper vs. lower panel). The definition of isochores is based on sliding windows of larger or smaller sizes (~1kb) and one could have expected that at the scale of an exon (~150 bp), the % of GC would be different from that of its hosting isochore. Similarly, it was not obvious that the composition bias of exons (150 bp) correlated with that of their hosting TADs (several dozen Kb). We also would like to point out that we integrated numerous experimental data from different origins (RNAseq, CLIPseq, ChIPseq, TAD annotations...) to generate a model integrating different scales (from ~100 nucleotides to several tens of Kbps) and to integrate different processes (splicing, chromatin organization, genome 1D and 3D organization). The analyses we performed based on nucleotide composition bias (from exons to genes, isochores, and TADs) provides a new piece of evidence of an interplay between the splicing process and the 1D and 3D genome organization (Fig. 7g).*

“2. A number of methodological issues need to be addressed more thoroughly, including:

- The definitions of the groups of GC-exons and AT-exons should be more rigorous, including the threshold values used for considering a flanking intron to be "short" or "long".”

*Response: We thank the Reviewer for raising these important issues. The definition of GC- and AT-exons was clarified (page 15, line 10). The threshold values were 49,3% of GC and 691 bp for the intron length.*

“- Similarly, the set of exons referred to as "control exons" should be more clearly defined. For example, on p. 13 of the methods section it is stated that "the control set of exons (typo: s is missing in the text) used corresponds to human exons annotated in FASTERDB." Is this the entire set of annotated human exons or a subset of exons randomly selected to match the number of exons contained in GC- and AT-exon sets that were being compared?”

*Response: The definition of “control exons” was clarified (page 15, line 30). The same set of control exons (labelled as CTRL exons) was used throughout the manuscript and figures.*

“- What is the overlap between the "U1-exons" and the "GC-exons" or the "AT-exons?" Likewise, how much overlap is there between the "U2-exons" and the GC- or AT-exon sets? “

*Response: About 28% of GC-exons and 14% of AT-exons were regulated by U1-related factors, while about 27% of AT-exons and 17% of GC-exons were regulated by U2-related factors. The manuscript was modified accordingly (page 7, line 22). It is also important to highlight that GC-exons were statistically more frequently regulated by three different U1-related factors, while AT-exons were statistically more frequently regulated by four different U2-related factors (Fig. 3g).*

“- Why were the datasets involving "spliceosome associated factors" from Figure 3 not considered as a part of Figure 1 to define the "GC-exon" and "AT-exon" sets? “

*Response: It was important to exclude the exons impacted by spliceosome-associated factors in early analyses (Figures 1 and 2) since the data in Figures 1 and 2 allowed us to generate hypotheses that we were able to validate using experimental datasets related to spliceosome-associated factors (Figure 3). If these datasets were used from the beginning, we would not have been able to reuse them to test our model.*

“- More generally, the authors initially used published datasets in which splicing factors had been knocked-down or overexpressed to choose the groups of "GC-exons" and "AT-exons." However, when they went on to look at the surrounding GC/AT environment in genes/isochores/TADS, why did they limit this analysis to this same set of ~7000 exons. Likely, many more exons are alternatively spliced, regulated by splicing factors, and fit the same criteria used to select the GC and AT exons even though they don't happen to be a part of the RNAseq datasets involving knockdown of select splicing factors. Could the authors look at an expanded set of GC-rich and AT-rich exons to analyze the GC content of their genomic environment? That way they could show that their assertion is not limited to these ~7000 exons but is a more general characteristic of GC-rich or AT-rich exons. They could then look at iCLIP data to evaluate if splicing factors are binding the expanded set of exons as predicted.”

*Response: We thank the Reviewer for this suggestion. To compare AT- and GC-rich exons independently of their regulation by auxiliary splicing factors, we selected human exons that share the same characteristics (GC composition and flanking intron size) as GC- or AT-exons but that are not regulated by the analyzed auxiliary splicing factors. These exons, labelled oGC- and oAT-exons, share the same properties than GC- and AT-exons, respectively, in terms of secondary structures at 5’ss, and number of 3’-related decoy sites (new Supplementary Fig. 9). Several (but not all) splicing factors regulating GC- or AT-exons (Fig.1d) were more frequently detected by ClIP analyses in the vicinity of oGC- or oAT-exons, respectively (new Supplementary Fig. 9f). However, the analysis of certain splicing factor ClIPs did not allow to conclude because the read density was identical when comparing oAT- and oGC-exons (see for example, SRSF7 on Supplementary Fig. 9f). This could be due to the fact that splicing factors can bind to RNAs in a splicing-unrelated manner. The fact that oGC- and oAT-exons were not in our lists of exons regulated by one of the 33 analyzed splicing factors, does not mean that these exons are not dependent on an non-analyzed splicing factor or a combination of splicing factors that can have redundant effects. Collectively, the analysis of oGC- and oAT-exons confirmed that nucleotide composition bias constrains specific splicing-related features and contributes to define U1- or U2-dependency. The manuscript was modified accordingly (page 12, line 16).*

“3. Key insights of the manuscript rely on predictions (RNA secondary structures for 5' ss in GC-rich exons, branchpoints associated with AT-rich exons) that have limited value in the absence of functional data, but that the authors raise to the level of conclusions present even in the abstract. It would greatly enhance the value of the manuscript if the authors would make an effort to experimentally test some of their predictions/conclusions. For example, could they assess the existence of secondary structures in one or two examples of GC-rich exons using minigenes, proving for example that disruption / compensatory mutations of predicted base pairing affects exon inclusion and that dependence on DDX5/17 is no longer there when the secondary structures are disrupted? Similar experiments could be designed to address the influence of the existence of potential branch sites in the inclusion of AT-rich exons. More generally, the authors could make use of recent branch point mapping efforts (e.g. Mercer et al 2015) to substantiate the real use of predicted branchpoints.”

*Response: Several publications have already shown that the negative effect of GC-rich structures on 5’ splice site recognition can be reversed by the helicase activity of DDX5 and DDX17 [3-5, 55], as now better highlighted in the Introduction (page 3, line 6) and in the Result section (page 7, line 27). Consequently, we decided to focus on the regulation at the 3’ ss level. To address the Reviewer’s concern, we selected from the analyzed RNAseq datasets three exons that were activated by SF1 and U2AF2, and we tested the effect of several AT-exon regulating factors such as PTBP1, MBNL1, hnRNPK, and TRA2 (Fig. 1d). As shown in the new Figure 4a, the selected exons were more excluded in the absence of both SF1 and U2AF2 (siU2, Fig. 4a) and the depletion of PTBP1, MBNL1, hnRNPK, or TRA2 had a selective effect on the different exons (Fig. 4a and 4b-d). Interestingly, the co-transfection of 2′-O-methylated antisense RNA oligonucleotides (AONs) targeting potential decoy BPs restored exon inclusion (Fig. 4b-d). For example, the depletion of PTBP1 or hnRNPK induced the exclusion of TD52L2 exon 4, which was reversed by the AON_TDP52L2 (Fig. 4b). These results showed that AONs can compensate the absence of AT-exon regulating splicing factors and are in agreement with a role of splicing factors in “filling up” or compensate a “surplus” of splicing signals (page 7, line 27 and see Discussion, paragraph starting page 11, line 32).*

*We tried to use BP’s annotation from experimental data. About 11% of the predicted BPs used in our work were found in the experimental data. This was expected and did not allow us to work with experimental BPs because these BPs were obtained from a limited number of samples, especially from “basal” experimental conditions in which many decoy sites are expected to be “masked”. It would be interesting to be able to identify alternative and decoy sites after depletion of different kind of splicing factors.*

“4. The connections between epigenetic marks / 3D organization and exon recognition are particularly weak. For example, are the histone marks that are more prevalent for the "GC-exons" a consequence of open chromatin related to the higher levels of transcription associated with these exons, or is it related to splicing regulation? The conclusions drawn from this panel are unclear— "Therefore, GC-exons and AT-exons…are embedded in different chromatin environments." What is the significance of this?”

*Response: We apologize for the lack of clarity in this part. Indeed, our objective was not to establish a particular relationship between histone marks and splicing of GC- or AT-exons. The objective of former Figure 5 (now Figure 6) was essentially to show that GC- and AT-exons were hosted by genes being differentially organized at the chromatin level, as supported by differential nucleosome positioning and the presence of different histone marks when comparing GC- and AT-exon hosting genes. We then decided to test whether GC- and AT-exons belong to different TADs, since each TAD is known to be composed of genes having the same chromatin organization. We added a paragraph in the manuscript to better explain this transition (page 9, line 13).*

“Additional points:

a) In Figure 1c, exons dependent on SRSF2, SRSF3, and hnRNPC were enriched in GC content, but they were excluded from the "GC-exon" set because their flanking introns were not shorter than controls. It would be interesting to compare this set to the GC-rich + short intron group, since this could help to discriminate between the role of GC-content and flanking intron size, as well as possibly provide potential insights as to why SRSF2, SRSF3 and hnRNPC-dependent exons display different features.”

*Response: As requested, we analyzed into more details splicing-related features of SRSF2-, SRSF3-, and hnRNPC- activated exons and we added Supplementary Fig. 10 to report the results. SRSF2-, SRSF3-, and hnRNPC-activated exons had intermediate values in terms of GC-content, MFE at 5’ss, and 3’-related decoy sites when compared with GC- and AT-exons (Supplementary Figure 10). It is therefore very difficult to answer to the Reviewer’s question but by addressing this concern, we can emphasize that each set of co-regulated exons had a combination of specific features. For example, SRSF2-activated exons had higher density of Gs, lower MFE values at 5’ss, and weaker 5’ss when compared with SRSF3- or hnRNPC-activated exons (Supplementary Figure 10a, b). Meanwhile, SRSF3-activated exons had higher density of Cs, a larger number of 3’ss-related decoy sites, larger exons and flanking introns when compared with SRSF2- or hnRNPC-activated exons (Supplementary Figure 10c, d). While we focused in the manuscript on two main families of exons (GC- vs AT-exons), each of them is likely composed of “sub-families” being characterized by a specific combination of features. It would be interesting in the future, to characterize the specific combination of features of each set of splicing-factor-regulated exons and to more deeply examine the interplay between combination of features and the precise nature of exon/gene nucleotide composition bias, which can be expected to be more complex than just based on the GC content. The manuscript was modified accordingly (page 12, line 29).*

“b) Several datasets examining SF3B1 knockdown are mentioned as being analyzed in the supplemental material, but the results of these analyses are not mentioned. Could the authors please explain this? “

*Response: We thank the Reviewer for raising this error. Our initial objective was to analyze as many splicing factors as possible. To ensure the biological relevance of our observations, we first compared the results obtained from different datasets for each splicing factor. In the vast majority of cases, the features of exons regulated by a given splicing factor were independent of the dataset used (e.g., cell line). One of the few exceptions was SF3B1 for which the properties of regulated exons differed from one dataset to another. Since we did not know whether this inconsistency was due to problems in one of the analyzed datasets, we preferred not to take SF3B1 into account in our analyses but we forgot to remove it from the Supplementary Table 1. This has been fixed.*

“c) Page 7, line 31. It is unclear what "In this setting…" in the beginning of the paragraph is referring to.”

*Response: “In this setting” was removed.*

d) Page 6: the sentence "the Py tract of GC-exons was similar to that of control exons" may be misleading, as Us provide higher binding affinity to U2AF2 than Cs and, for the same reason, the conclusion that "the analysis of U2AF2 CLIP-Seq datasets revealed a higher U2AF2-related signal upstream of AT-exons, as compared to GC-exons (Fig. 2h)" is not unexpected.

*Response: We thank the Reviewer for this pertinent comment. By "the Py tract of GC-exons was similar to that of control exons" we meant that the density of pyrimidines in the Py tract was the same when comparing GC-exons with control exons. This does not mean that C- and U-rich Py tracts are functionally identical as now stated in the revised manuscript (page 6, line 8 and page 6, line 28). Although the results obtained from the U2AF2-ClIP analyses were not unexpected, it was important to perform these analyses to present supporting experimental data.*

**Second round of review**

**Reviewer 2**

The authors have satisfactorily addressed the issues raised in my previous report and I am happy to recommend publication of the manuscript in Genome Biology.
